# Rice Carbohydrate-Binding Malectin-Like Protein, OsCBM1, Contributes to Drought-Stress Tolerance by Participating in NADPH Oxidase-Mediated ROS Production

**DOI:** 10.1186/s12284-021-00541-5

**Published:** 2021-12-07

**Authors:** Xiu-Qing Jing, Wen-Qiang Li, Meng-Ru Zhou, Peng-Tao Shi, Ran Zhang, Abdullah Shalmani, Izhar Muhammad, Gang-Feng Wang, Wen-Ting Liu, Kun-Ming Chen

**Affiliations:** 1 State Key Laboratory of Crop Stress Biology in Arid Area, College of Life Sciences, Northwest A&F University, Yangling, 712100 Shaanxi China; 2 Department of Biology, Taiyuan Normal University, Taiyuan, 030619 Shanxi China

**Keywords:** Malectin-like domain-containing protein, OsCBM1, Drought tolerance, OsRbohA, OsRacGEF1, ROS production, Rice (*Oryza sativa*)

## Abstract

Carbohydrate-binding malectin/malectin-like domain-containing proteins (CBMs) are a recently identified protein subfamily of lectins that participates various functional bioprocesses in the animal, bacterial, and plant kingdoms. However, little is known the roles of CBMs in rice development and stress response. In this study, *OsCBM1*, which encodes a protein containing only one malectin-like domain, was cloned and characterized. OsCBM1 is localized in both the *endoplasmic reticulum* and plasma membrane. Its transcripts are dominantly expressed in leaves and could be significantly stimulated by a number of phytohormone applications and abiotic stress treatments. Overexpression of *OsCBM1* increased drought tolerance and reactive oxygen species production in rice, whereas the knockdown of the gene decreased them. OsCBM1 physically interacts with OsRbohA, a NADPH oxidase, and the expression of *OsCBM1* in *osrbohA,* an *OsRbohA*-knockout mutant, is significantly downregulated under both normal growth and drought stress conditions. Meanwhile, OsCBM1 can also physically interacts with OsRacGEF1, a specific guanine nucleotide exchange factor for the Rop/Rac GTPase OsRac1, and transient coexpression of *OsCBM1* with *OaRacGEF1* significantly enhanced ROS production. Further transcriptome analysis showed that multiple signaling regulatory mechanisms are involved in the OsCBM1-mediated processes. All these results suggest that OsCBM1 participates in NADPH oxidase-mediated ROS production by interacting with OsRbohA and OsRacGEF1, contributing to drought stress tolerance of rice. Multiple signaling pathways are likely involved in the OsCBM1-mediated stress tolerance in rice.

**Supplementary Information:**

The online version contains supplementary material available at 10.1186/s12284-021-00541-5.

## Background

Carbohydrate-binding malectin domain or malectin-like domain-containing proteins (CBMs) are a recently identified protein subfamily of lectins that occur across the animal, bacterial, and plant kingdoms (Schallus et al. 2008; Bellande et al. 2017). CBMs, initially found in bacteria and fungi, are non-catalytic domains frequently appended to glycoside hydrolases that degrade plant cell wall polysaccharides (Cantrarel et al. 2009). Thereafter, they were identified in all taxonomic kingdoms. Malectin (PF11721) binds with a di-glucose within the *endoplasmic reticulum* (ER) and NMR-based ligand-screening studies have shown binding of the protein to maltose and related oligosaccharides. Accordingly, the protein has been designated “malectin”, and its endogenous ligand was determined to be Glc2-high-mannose N-glycan in animal cells (Schallus et al. 2008). Malectin-like (PF12819) is a carbohydrate-binding protein of the ER and this domain has been found existing in a number of plant receptor kinases (Zhang et al. 2007; Boisson-Dernier et al. 2011).

The well-described CBMs in plants are malectin-like receptor kinases, also known as *Catharanthus roseus* receptor-like kinase-like proteins (CrRLK1Ls), which belong to a new receptor-like kinase (RLK) subfamily and are involved in many different plant functional processes ranging from cell-wall integrity to immunity (Franck et al. 2018). All members of the CrRLK1L protein family in plants share a similar domain structure with a malectin-like domain, a transmembrane domain, and an intracellular Ser and Thr kinase domain (Boisson-Dernier et al. 2011; Lindner et al. 2012; Galindo-Trigo et al. 2016). In *Arabidopsis*, the CrRLK1L family contains 17 members and 10 are characterized as having important roles in cell growth, plant morphogenesis, reproduction, immunity, hormone signaling, and stress responses (Boisson-Dernier et al. 2011; Kessler et al. 2015; Yeh et al. 2016; Feng et al. 2018; Franck et al. 2018). FERONIA (FER) is the best characterized CrRLK1L family protein. It is a key positive regulator for polar growth and cell-wall integrity. The *fer* mutant, loss-of-function plants for *FER*, are semi-dwarf and the tip-growing cells, such as trichomes and root hairs, in *fer* plants are affected (Guo et al. 2009; Duan et al. 2010). The *fer* mutant plants also display box-shaped epidermal cells (Li et al. 2015). FER is also essential for pollen tube reception and in mechanical signaling transduction in *Arabidopsis* by interacting with reactive oxygen species (ROS) and Ca^2+^ (Kessler et al. 2015; Shih et al. 2014; Nissen et al. 2016). Although FER is required for pollen tube reception in the female gametophyte, its closest homologs, ANXUR1 (ANX1) and ANXUR2 (ANX2), function in the maintenance of pollen tube integrity during polarized tip growth and the premature bursting of pollen tubes in *anx1* or *anx2* mutants leads to male sterility (Boisson-Dernier et al. 2009; Miyazaki et al. 2009). FER also acts as a negative regulator of abscisic acid (ABA) signaling by activating ABI2 phosphatase (Yu et al. 2012). More recently, it was reported that FER acts as a damage sensor to maintain cell-wall integrity during salt stress through Ca^2+^ signaling (Feng et al. 2018). Additionally, FER positively influences pathogen-associated molecular pattern (PAMP)-triggered immunity (PTI) in association with BRASSINOSTEROID INSENSITIVE 1-ASSOCIATED RECEPTOR KINASE 1 (BAK1) (Stegmann et al. 2017; Franck et al. 2018). Another malectin-like receptor kinase IOS1 also positively regulates plant immunity by association with BAK1 (Yeh et al. 2016). ANX1 and ANX2 also negatively regulate both the cell surface-resident pattern recognition receptor (PRR)- and intracellular nucleotide-binding domain leucine-rich repeat protein (NLR)-mediated immunity in association with FLAGELLIN-SENSING2 (FLS2) and BAK1 (Mang et al. 2017). In rice, two CrRLK1L homolog genes, *OsCrRLK1L2* and *OsCrRLK1L3*, were found to be antagonistically upregulated in a diurnal and nocturnal manner, respectively (Nguyen et al. 2015). Another rice CrRLK1L homolog gene, *OsCrRLK1L15*, may participate in the salt stress response (Udomchalothorn et al. 2014). In strawberries, a genome-wide analysis indicated that malectin-like receptor kinases in this species are associated with fruit ripening and abiotic stress responses (Zhang et al. 2016).

However, CBMs are diverse in structure according to the domain composition, and not only CrRLK1L-type proteins exist in plants. In a recent study, a total of 69 members of CBMs were identified in *Arabidopsis* and among them, 59 members contained a cytoplasmic kinase domain (Bellande et al. 2017), which is greater than the 17 *Arabidopsis* CrRLK1L genes previously identified (Boisson-Dernier et al. 2011). Among these *Arabidopsis* CBMs, nine members do not have a kinase domain but have leucine-rich repeat (LRR) motifs in amino acid sequences, whereas one member only contains one malectin-like domain in the N-terminal (Bellande et al. 2017). In strawberries, 62 CBM members were identified and some members contain only one malectin domain (Zhang et al. 2016). In the rice genome, 16 genes were identified as CrRLK1L homologs in an early study (Nguyen et al. 2015). However, according to our recent study, a total of 84 *CBMs* genes were identified in the rice genome, with 67 encoding malectin/malectin-like receptor-like kinases (MRLKs) and 17 encoding malectin/malectin-like receptor-like proteins (MRLPs) (Jing et al. 2020). These MRLPs do not have a kinase domain but have one malectin or malectin-like domain in their protein structure.

In the present study, a CBM gene (LOC_09g19380), named *OsCBM1*, was cloned and characterized in rice. OsCBM1 only contains a malectin-like domain in its structure and was denoted as OsMRLP15 in our previous study (Jing et al. 2020). OsCBM1 is located at both the ER and PM. Its transcripts are dominantly expressed in leaves of rice and can be greatly stimulated by a number of phytohormonal application and abiotic stresses. Overexpression of *OsCBM1* enhanced plant tolerance to drought, whereas its downexpression reduced the tolerance. OsCBM1 participates in ROS production by interacting with OsRbohA and OsRacGEF1, therefore contributing to drought tolerance of rice. The results obtained here proposed a novel signaling mode for CBM proteins functioning in stress tolerance of plants.

## Results

### OsCBM1 is Only a Malectin-Like Domain Containing Protein Located Both at the ER and PM and Functions in the Stress Response of Rice

**Fig. 1:**
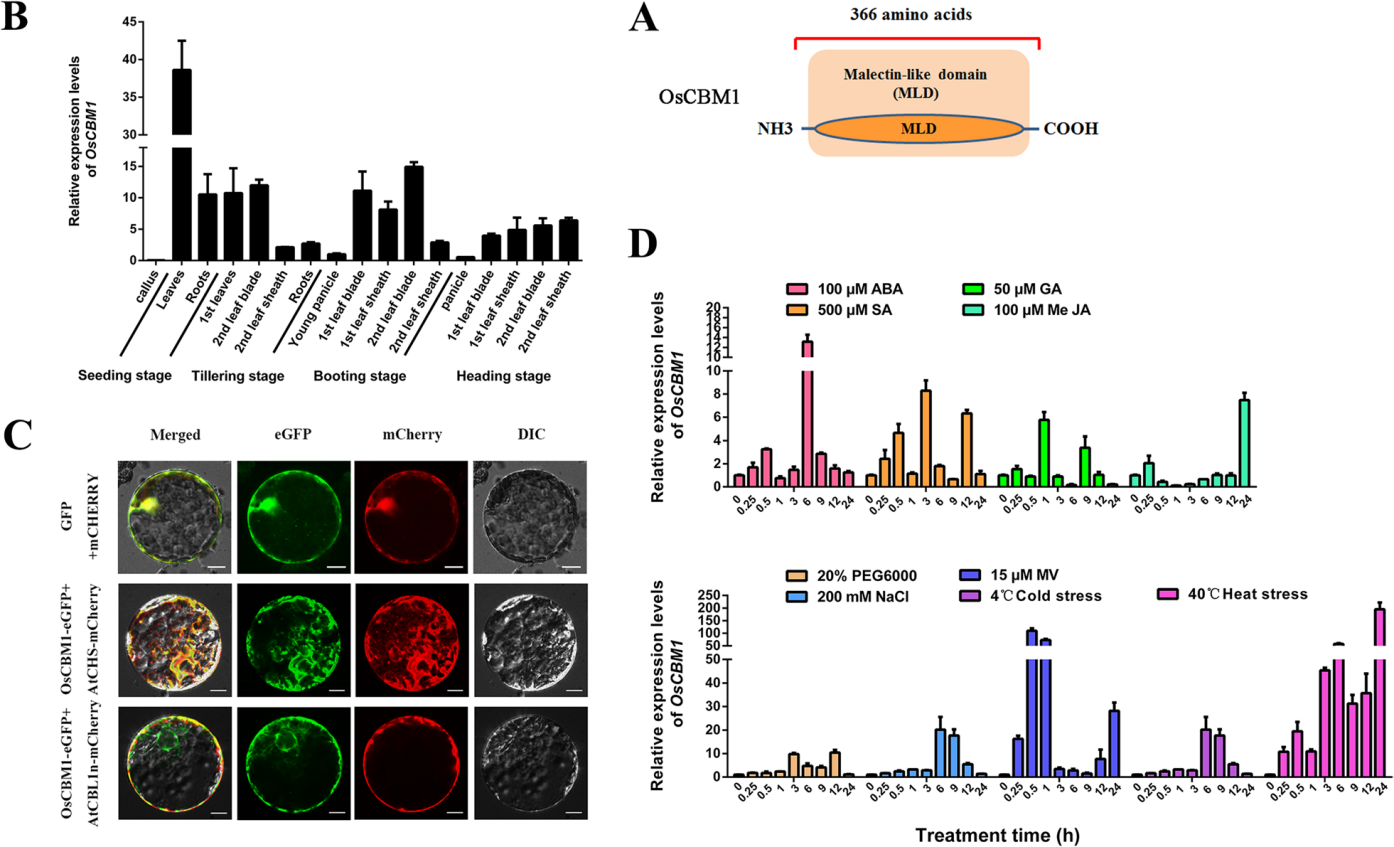
Tissue-specific expression patterns and subcellular localization of OsCBM1 in rice. **A** Protein structure of *OsCBM1*, showing it is an only malectin-like domain-containing protein. **B** Tissue-specific expression pattern of *OsCBM1*. The wild-type (WT, Nipponbare) of rice plants were allowed to grow in paddy fields and the different plant organs or tissues at various developmental stages, namely seedling, tillering, booting, and heading stages, were collected. After harvest, the developmental and tissue-specific expression patterns of *OsCBM1* were assessed with qRT-PCR, using *OsActin1* as the internal control. **C** Analysis of the subcellular localization of OsCBM1 using a *Nicotiana benthamiana* protoplast transient transformation system, showing both the *endoplasmic reticulum* (ER) and plasma membrane (PM) localization of OsCBM1. AtCBL1n-mCherry served as a marker for PM protein localization and AtCHS-mCherry was used as a marker for ER protein localization. Bars = 10 μm. **D** Inducible expression profiles of *OsCBM1* in response to hormone treatments and abiotic stresses. Healthy and uniform rice seedlings (WT, Nipponbare) were selected and grown in a hydroponic solution. Two-week-old seedlings were exposed to various abiotic stresses and phytohormones, including ABA (100 μM), SA (500 μM), GA (50 μM), MeJA (100 μM), dehydration (20% PEG6000), salt (200 mM NaCl), oxidative (15 μM MV), cold (4 °C) and heat (40 °C). The whole leaf blades from the plants were harvested at 0, 0.25, 0.5, 1, 3, 6, 9, 12, and 24 h intervals after treatments. Relative expression levels of *OsCBM1* were analyzed with qRT-PCR using *OsActin1* as the internal control. Data are means of three biological replicates (n = 9)

In our previous study, a total of 84 *CBMs* genes were identified in the rice genome, with 67 encoding malectin/malectin-like receptor-like kinases (MRLKs) and 17 encoding malectin/malectin-like receptor-like proteins (MRLPs) (Jing et al. 2020). Among them, OsMRLP15 (LOC_09g19380) was found to only contain a carbohydrate-binding malectin-like domain in the protein structure (Fig. 1A), and thus, was renamed herein as OsCBM1. As shown in Fig. 1B, *OsCBM1* is expressed in the entire rice plant and the most dominant transcripts occur in leaves rather than in roots or panicles during the whole development stages examined. The subcellular localization analysis using a *Nicotiana benthamiana* leaf transient transformation system showed that OsCBM1 is located at both the ER and PM in cells (Fig. 1C). The *OsCBM1-GFP* construct was expressed both with the ER marker *AtCHS* (Saslowsky and Winkel-Shirley 2001, 2006) and the PM marker *AtCBL1n* (Batistic et al. 2010), which were fused to the *mCherry* reporter gene, respectively (Fig. 1C).

Additionally, *OsCBM1* was highly sensitive to a number of hormonal treatments and environmental abiotic stresses. As shown in Fig. 1D, the transcriptional expression of the gene was stimulated by exogenous abscisic acid (100 μM ABA), salicylic acid (500 μM SA), gibberellic acid (50 μM GA), and methyl jasmonic acid (100 μM MeJA) applications (Fig. 1D). The expression of the gene was also induced by dehydration (20% PEG6000), salt (200 mM NaCl), oxidative stress (15 μM methyl viologen, MV), cold (4 °C), and heat (40 °C) stress treatments (Fig. 1D), particularly by MV and heat stresses, suggesting that *OsCBM1* is a stress-responsive gene and might play crucial roles in abiotic stress tolerance of rice.

### Differential Expression of *OsCBM1* Affected the Agronomic Traits of Rice Plants

**Fig. 2:**
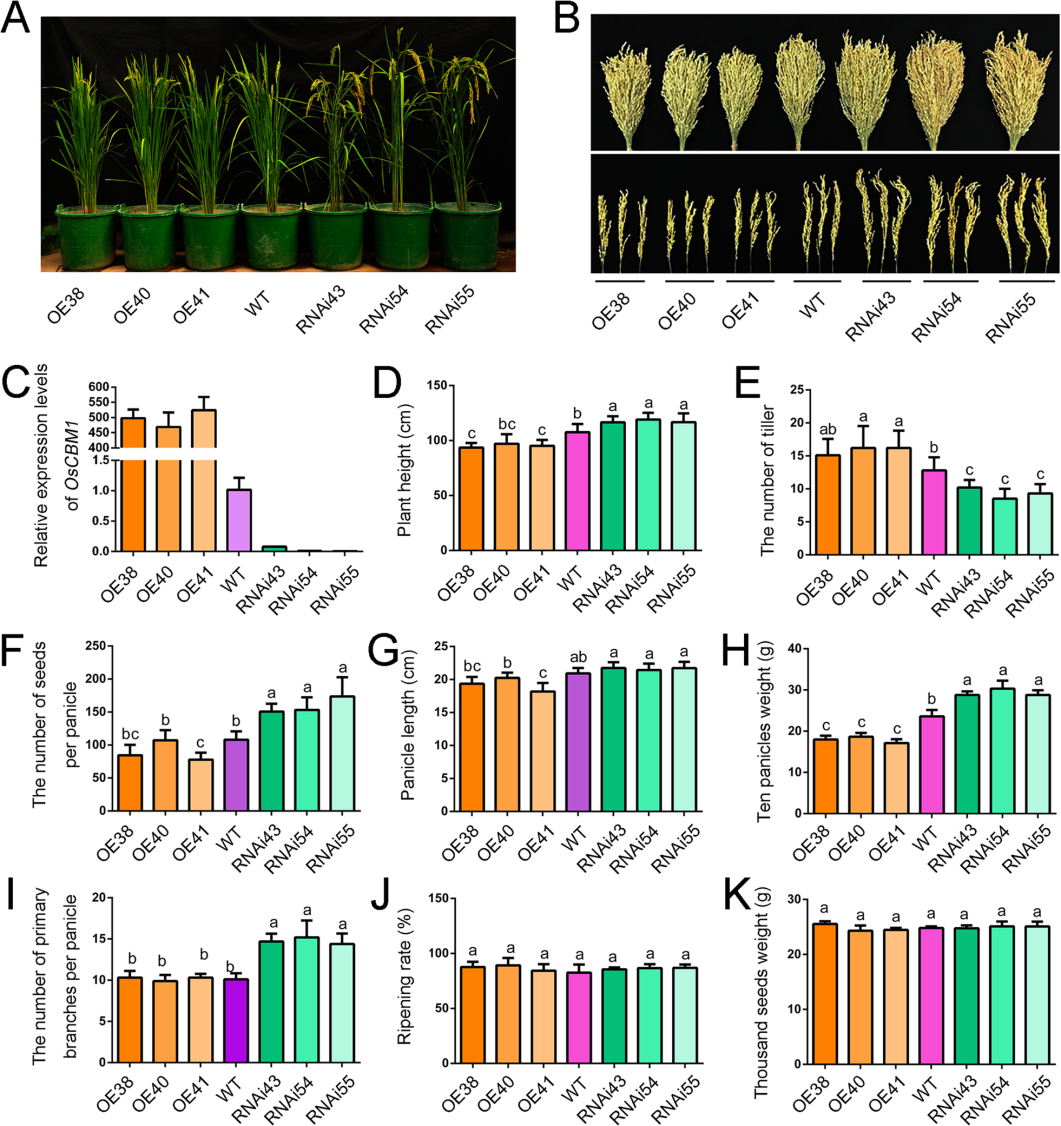
Differential expression of *OsCBM1* affected the morphology of rice plants and their agronomic traits. **A** Gross morphology of wild-type (WT, Nipponbare) and the *OsCBM1*-overexpressed (OE) or -RNA interference (RNAi) transgenic plants at heading stages. **B** Harvested panicle morphology of the different plant lines, the top picture showing the morphology of 20 panicles bundled together. **C** Transcriptional levels of *OsCBM1* in the different plant lines at the heading stages detected by qRT-PCR using *OsActin1* as the internal control. **D** Plant height of the plants at the ripening stage. **E** The tiller numbers of the different plant lines. **F**–**K** Yield-related parameters between the WT and *OsCBM1*-transgenic plants. The plants were grown in pots filled with paddy soil under natural growth condition and at least 30 plants were used for the calculation of the agronomic traits. Bars annotated with different letters represent values that are significantly different (*p* ≤ 0.05) according to a one-way ANOVA

To obtain further insights into the OsCBM1 function, we generated transgenic plants, including the gene overexpressing (OE) and RNA interference (RNAi) lines by *Agrobacterium*-mediated transformation with rice calli from *Oryza sativa* L. subsp. *japonica* Nipponbare. Three independent lines with different transcription levels of *OsCBM1* for both the OE and RNAi plants (T2 generation) were selected for further study (Fig. 2A, C, and Additional file 1: Fig. S1). As compared to the wild-type (WT), the OE plants showed little alteration in plant height (Fig. 2D), number of tillers (Fig. 2E), number of seeds per panicles and panicle length (Fig. 2B, F, G). Only the ten-panicle weight exhibited a marked decrease in the OE plants compared with that of the WT (Fig. 2H). In contrast, the knockdown of *OsCBM1* increased plant height (Fig. 2D), numbers of seeds per panicle (Fig. 2F), primary branches per panicle (Fig. 2I), and the ten-panicle weight (Fig. 2H) but decreased the number of tillers (Fig. 2E) as compared to that of the WT. However, the seed ripening rate and thousand seed weight were not affected in either the OE plants or the RNAi plants (Fig. 2J, K).

### OsCBM1 Participates in Drought Tolerance and ROS Production of Plants by Physically Interacts with OsRbohA

**Fig. 3:**
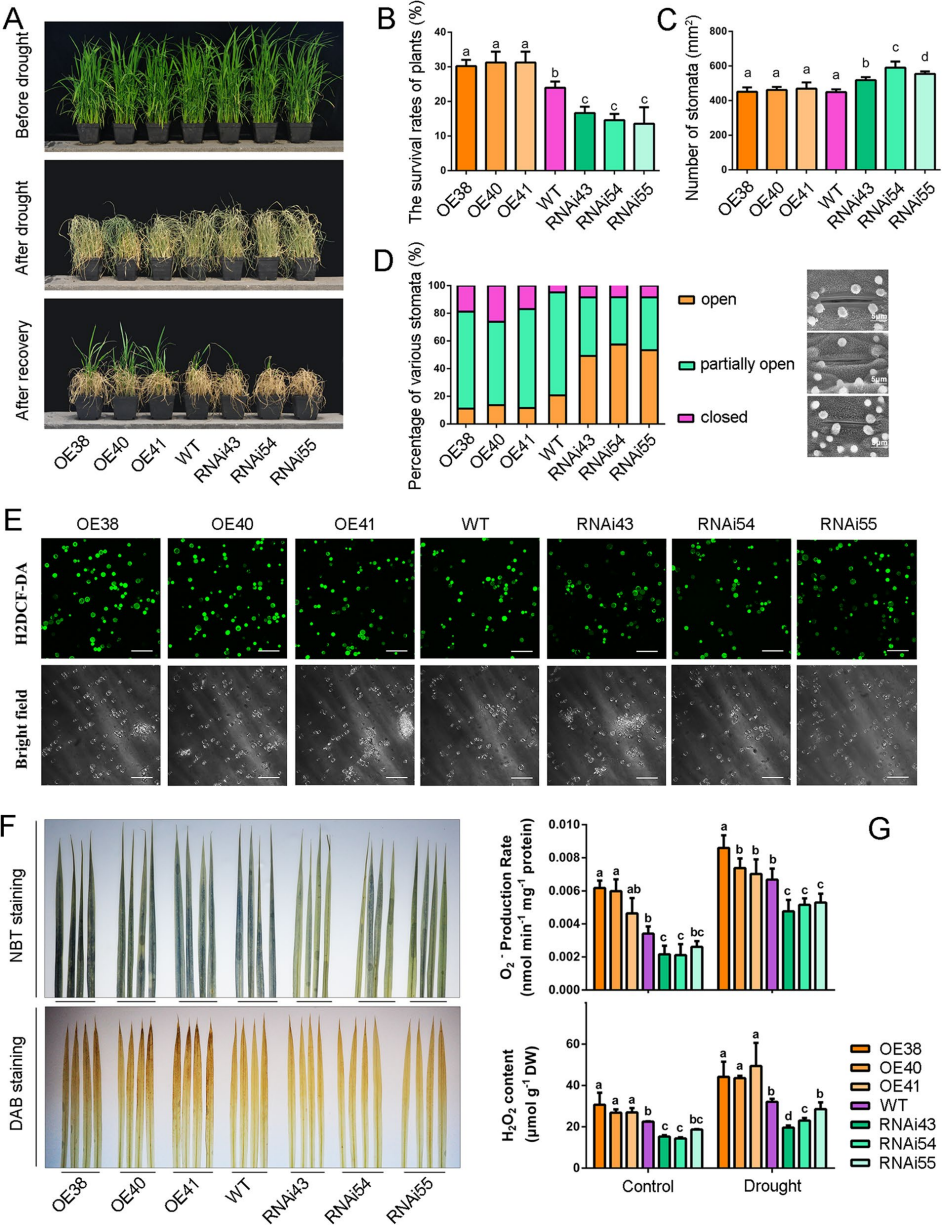
OsCBM1 positively regulates drought tolerance and reactive oxygen species (ROS) production of rice. **A** Phenotypes of wild-type (WT), *OsCBM1*-overexpressing (OE), and RNA interference (RNAi) plants before drought treatment (the top pictures), after drought (the middle pictures, without watering for 5 d), and re-watering for 14 d (the bottom pictures). Four-week-old plants were used for drought treatments. **B** Survival rates of the different plant lines after the drought. At least 50 plants were used in each survival statistics experiment. Data are means ± SD from three independent biological replicates. **C** The numbers of stomatal apparatuses in the abaxial epidermis of flag leaves between the different plant lines under normal growth conditions. At least 2000 stomata in 30 microscopy pictures from different leaves were used to calculate values for each line of plants. **D** Percentage of various stomatal apertures in the abaxial epidermis of flag leaves between the different plant lines. For each type of plant, at least 500 stomata apparatuses from different plant leaves were calculated. **E** Visualization of ROS levels in the protoplasts isolated from the various plant lines. Cytoplasmic ROS were detected with H_2_DCF-DA fluorescence. Bars = 50 μm. **F** H_2_O_2_ and O_2_^−^ histochemical analyses in leaves of the 6-week-old plants under normal growth conditions stained with 1% 3,3′-diaminobenzidine tetrachloride (DAB) or 0.1% nitroblue tetrazolium (NBT), respectively. G, Contents of H_2_O_2_ (μmol g^−1^ DW), and production rate of O_2_^−^ (nmol min^−1^ mg^−1^ protein) in leaves of the 6-week-old plants under normal growth and drought stress conditions. Bars annotated with different letters represent values that are significantly different (*p* ≤ 0.05) according to a one-way ANOVA

Because the *OsCBM1* gene exhibited markedly stress-inducible characteristics in transcriptional expression, as described above, we next examined the tolerance of the gene transgenic plants to drought. The results showed that the OE plants of *OsCBM1* exhibited greater tolerance to drought, whereas the RNAi plants were more sensitive to the stress (Fig. 3A). The survival rates of the plants under drought were higher in the OE lines but lower in the RNAi lines compared with that of the WT (Fig. 3B). Further study on the characteristics of the stomatal apparatus of the leaves between the different types of plants showed that, the *OsCBM1*-RNAi lines possessed greater numbers of stomata per leaf area (Fig. 3C) and a higher percentage of opening stomata (Fig. 3D), whereas, most of the stomata in the leaves of the OE lines stayed partially opening (Fig. 3D), implying that water will be more easily lost in the RNAi plants and therefore, decreased the tolerance of the plants to drought. However, the water use efficiency (WUE) of the plants retained no changes between the WT, OE, and RNAi lines plants both under normal growth and drought stress conditions and even drought stress seemed to decreased the WUE in all types of plants (Additional file 2: Fig. S2). Under normal growth conditions, there were no significant differences in the rates of photosynthesis, transpiration, and the conductance of stomata among the WT, OE, and RNAi plants. However, under drought conditions, these three indexes were highest in RNAi plants, followed by WT plants, with the lowest values in OE plants, although no significant differences were observed between some plant lines (Additional file 2: Fig. S2).

Meanwhile, we examined the ROS production of the genetically transgenic plants and found that ROS (H_2_O_2_ and O_2_^−^) production was much more highly exhibited in both protoplasts and leaves of the OE plants but was markedly lower in those of the RNAi plants compared with that of the WT, even under normal growth condition. As shown in Fig. 3E, more protoplasts isolated from the OE line plants exhibited bright fluorescence of 2,7-dichlorodihydrofluorescein dilacerate acetyl ester (H_2_DCF-DA) as compared to those from the RNAi plants, indicating that the intracellular ROS levels were higher in the OE plants than in the RNAi plants. The results from the leaves of both the nitro blue tetrazolium (NBT) and 3,3′-diaminobenzidine tetrachloride (DAB) staining, which represented the release levels of H_2_O_2_ and O_2_^−^ in apoplastic spaces, respectively, demonstrated greater ROS production in the OE plants than in the RNAi plants because the OE leaves stained darker than did the RNAi ones (Fig. 3F). The stoichiometric analysis further revealed that the OE plants possessed higher leaf H_2_O_2_ content and O_2_^−^ production rate than did the RNAi plants both under normal growth and drought-stress conditions, although drought stress enhanced ROS production in both the OE and RNAi plants (Fig. 3G). These results suggested that the functions of OsCBM1 in drought tolerance of rice might be involved in the regulation of the protein in ROS production.

**Fig. 4:**
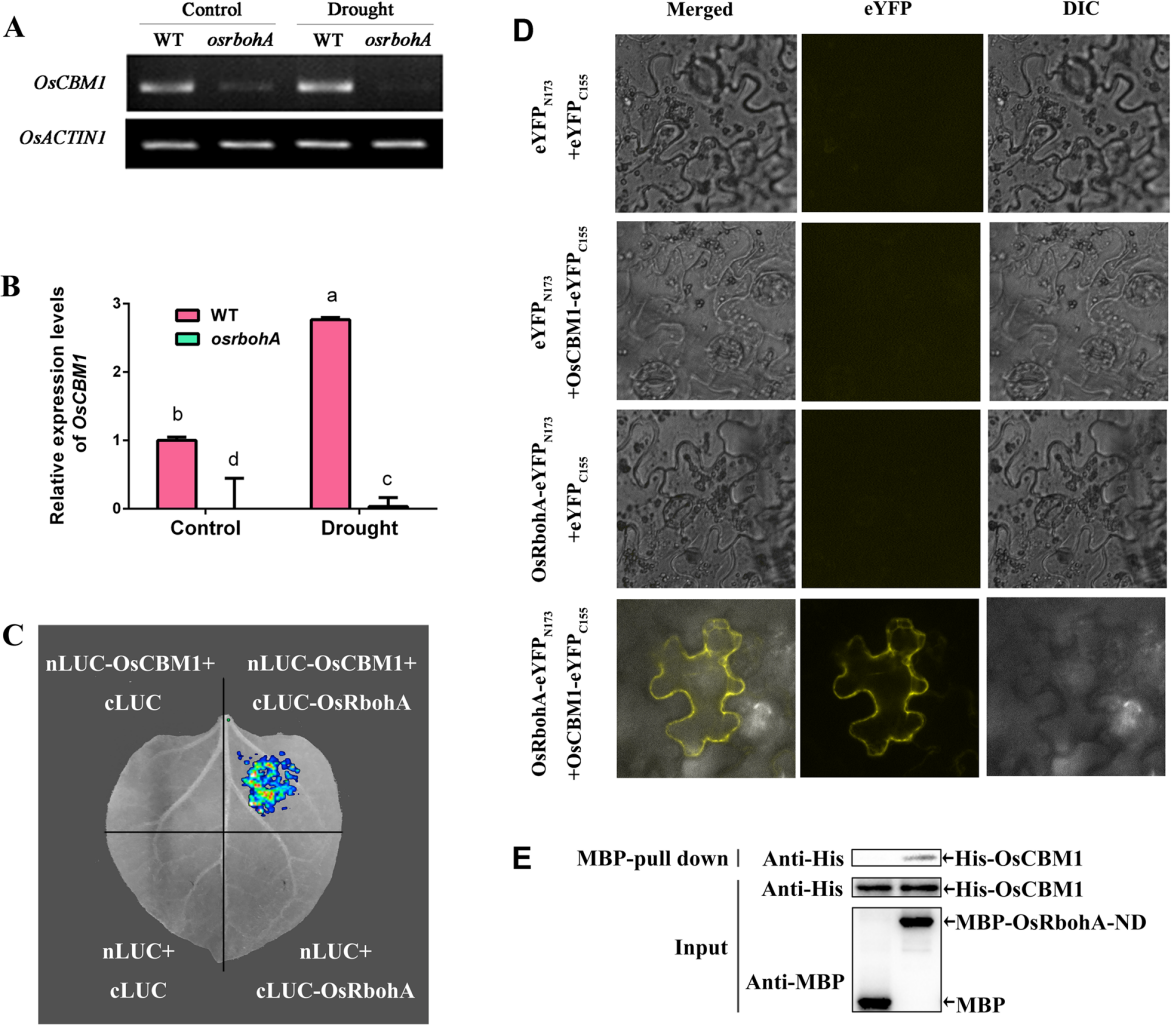
OsCBM1 interacts with OsRbohA and showed very high coexpressions with OsRbohA. **A** Expression levels of *OsCBM1* in the WT and *osrbohA* mutant according to a semi-quantitative RT-PCR experiment. **B** Expression levels of *OsCBM1* in the WT and *osrbohA* mutant by qRT-PCR experiment with *OsActin1* as the internal control. The leaves from 6-week-old plants under both control and drought were used for the determination. Data are means of three biological replicates (n = 12). Bars annotated with different letters represent values that are significantly different (*p* ≤ 0.05) according to a one-way ANOVA analysis. **C** Firefly luciferase complementation imaging (LCI) assay, showing the interaction of OsCBM1 with OsRbohA in vivo. **D** Bimolecular fluorescence complementation (BiFC) assay, showing the interaction of OsCBM1 with OsRbohA in vivo*.* **E** MBP-pull down assay, showing the interaction of OsCBM1 with OsRbohA-ND (coding 360 amino acid residues at the N-terminal) in vitro

Since the plasma membrane NADPH oxidase, also known as respiratory burst oxidase homologs (Rbohs), play vital roles in plant stress tolerance (Hu et al. 2020a, b; Shi et al. 2020) and OsRbohA, a key NADPH oxidase for ROS production, functions in the developmental regulation and drought-stress tolerance of rice (Wang et al. 2016), we then tested whether the functions of OsCBM1 in drought tolerance and ROS production are related to OsRbohA. As shown in Fig. 4, the transcriptional expression levels of *OsCBM1* were greatly downregulated in *osrbohA,* an *OsRbohA*-knockout mutant, as compared to the WT, under both normal growth and drought stress conditions (Fig. 4A, B), implying a close relationship between OsCBM1 and OsRbohA in biological functions. We further examined the interaction between OsCBM1 and OsRbohA at the molecular level. Firefly luciferase complementation imaging (LCI) assay were firstly used to examine the interaction and a fluorescent signal was detected in *Nicotiana benthamiana* leaves when cLuc-*OsRbohA* was coexpressed with nLuc-*OsCBM1* (Fig. 4C), indicating the interactive relationship between OsCBM1 and OsRbohA in vivo. Moreover, bimolecular fluorescence complementation (BiFC) assay was performed to verify the interaction between OsCBM1 and OsRbohA and the existence of eYFP fluorescence signal strongly suggests the interaction between OsCBM1 and OsRbohA in vivo (Fig. 4D)*.* Then MBP-pull down assay was performed to clarify the interaction of OsCBM1 and OsRbohA-ND (coding 360 amino acid residues at the N-terminal) in vitro. The His-tagged OsCBM1 could be pulled down by MBP-tagged OsRbohA, clearly showing that OsRbohA-ND was directly associated with OsCBM1 in vitro (Fig. 4E). All these results suggest that OsCBM1 physically interacts with OsRbohA and therefore, positively mediates ROS production and drought tolerance in rice.

### OsCBM1 Physically Interacts with OsRacGEF1

**Fig. 5:**
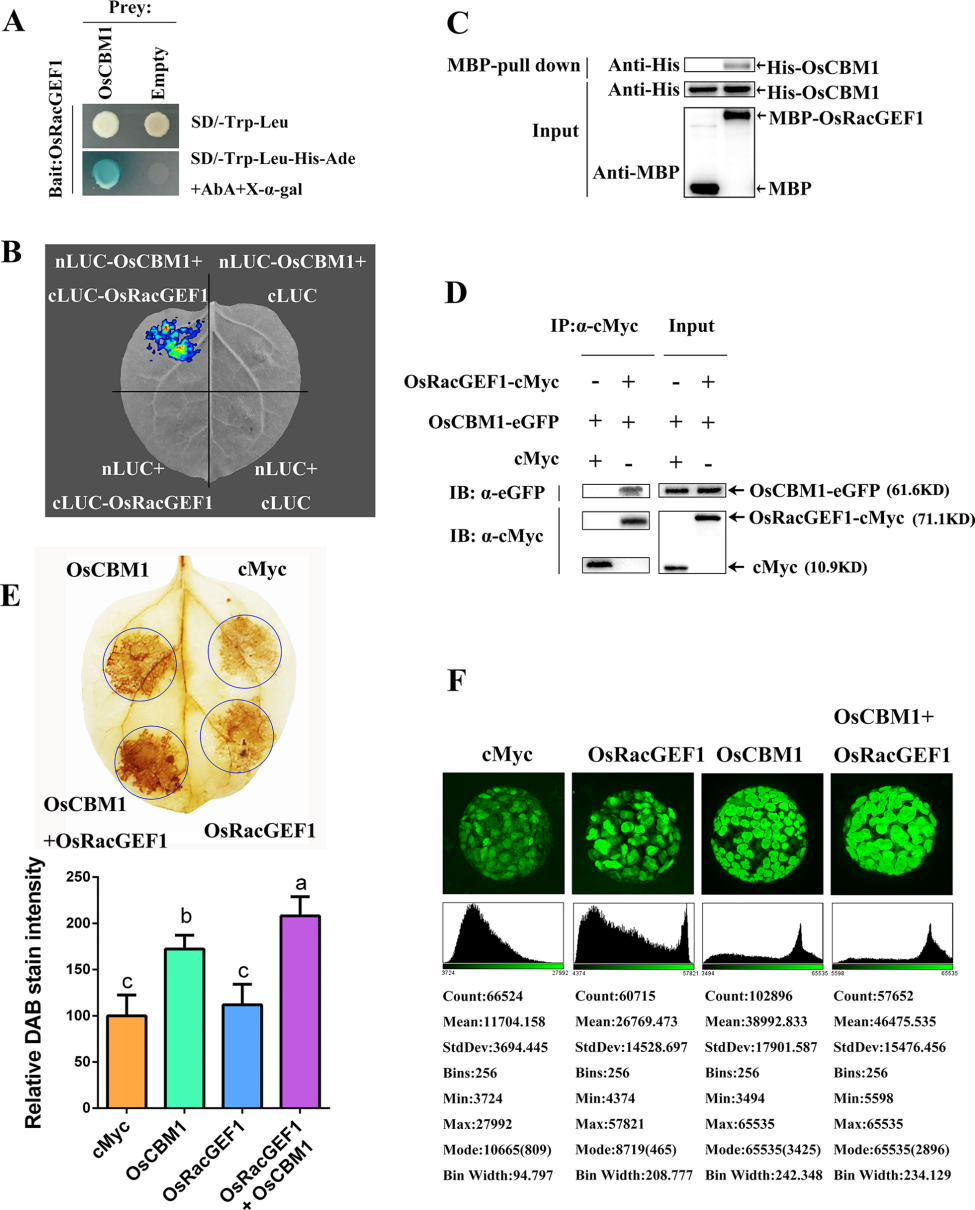
OsCBM1 interacts with OsRacGEF1 and their coexpression enhanced reactive oxygen species (ROS) production. **A** Split-ubiquitin yeast two-hybrid assays of the “bait” pGBKT7-OsRacGEF1 with the “prey” pGADT7-OsCBM1. **B** Firefly luciferase complementation imaging (LCI) assay. **C** MBP-pull down assay, showing the interaction of OsCBM1 with OsRacGEF1 in vitro. **D** Co-immunoprecipitation (Co-IP) assay, showing the physical interaction of OsCBM1-eGFP with OsRacGEF1-6 × cMyc in vivo. **E** Transient coexpression of OsCBM1 and OsRacGEF1 in the leaves of *Nicotiana benthamiana*. The 3,3′-diaminobenzidine (DAB)-stained *N. benthamiana* leaves were transiently transformed with cMyc (P_35S_-cMyc), OsCBM1 (P_35S_-OsCBM1), OsRacGEF1 (P_35S_-OsRacGEF1), and their combination, respectively. The DAB staining intensity in situ ROS levels of agroinfiltrated *N. benthamiana* leaves in each treatment was calculated based on the stain intensity of the control cMyc. Bars annotated with different letters represent values that are significantly different (*p* ≤ 0.05) according to a one-way ANOVA. **F** Detection of ROS production by H_2_DCFDA fluorescent probe in *N. benthamiana* protoplasts isolated from the leaves of *N. benthamiana* agroinfiltrated by different vectors. Bars = 10 μm. The intensity of fluorescent signals was calculated with ImageJ 1.8.0 software and presented with scatter diagrams (the bottom images in **F**)

Because OsRacGEF1 functions in the binding of OsRac1 to OsRbohs to regulate ROS production in rice (Wong et al. 2007; Akamatsu et al. 2013; Kawasaki et al. 2017), we next tested whether OsRacGEF1 participated in the OsCBM1-mediated ROS production. At first, we used the split-ubiquitin yeast two-hybrid assays to test the interaction between OsCBM1 and OsRacGEF1 and the results showed that OsCBM1 could interact with OsRacGEF1 in yeast cells (Fig. 5A). Next, LCI assay were conducted to examine the interaction again and a strong fluorescent signal was detected in *N. benthamiana* leaves when cLuc-*OsRacGEF1* was coexpressed with nLuc-*OsCBM1* (Fig. 5B), thereby further indicating the interactive relationship between OsCBM1 and OsRacGEF1 in vivo. Thereafter, an MBP-pull down assay was performed to clarify the interaction of OsCBM1 and OsRacGEF1 in vitro. The His-tagged OsCBM1 could be pulled down by MBP-tagged OsRacGEF1, clearly showing that OsRacGEF1 was directly associated with OsCBM1 in vitro (Fig. 5C). The Co-IP experiment further showed that OsCBM1 physically interacts with OsRacGEF1 because a clear band of OsCBM1-eGFP was observed in the OsRacGEF1-6 × cMyc immunoprecipitates when the eGFP-tagged OsCBM1 was co-expressed with OsRacGEF1-6 × cMyc in *N. benthamiana* leaves (Fig. 5D). Additionally, OsCBM1 and OsRacGEF1 were co-located in subcellular compartments because the fluorescent signals overlapped in both the *N. benthamiana* epidermal cells and protoplasts (Additional file 3: Fig. S3). Interestingly, a lack of OsCBM1 appeared to affect the subcellular localization of OsRacGEF1 to PM because the fluorescent signals of OsRacGEF1 in PM was weakened in the *OsCBM1*-RNAi54 transgenic plants compared with that of the WT (Additional file 4: Fig. S4).

### Transient Coexpression of OsCBM1 with OsRacGEF1 Enhanced ROS Production

Furthermore, we determined whether the interaction of OsCBM1 with OsRacGEF1 and OsRbohA contributes ROS production by using a transient co-expression system. As expected, transient co-expression of *OsCBM1* and *OsRacGEF1* greatly enhanced ROS production in *N. benthamiana* leaves (Fig. 5E). Deeply stainings were observed in the areas of both *OsCBM1* and *OsRacGEF1* expression and the co-expression of the two genes exhibited higher staining intensity, as compared to their expression alone. These results strongly suggested that OsCBM1 positively contributes ROS production by interacting with OsRacGEF1. The co-expression of OsCBM1 with OsRbohA in *N. benthamiana* can also enhance ROS production in *N. benthamiana* leaves (Fig. 5F), further suggesting that the OsCBM1-mediated ROS production might be also closely related to OsRbohA.

### Downregulation of *OsCBM1* Affected the Expression of Global Genes Related to Plant Stress Tolerance

**Fig. 6:**
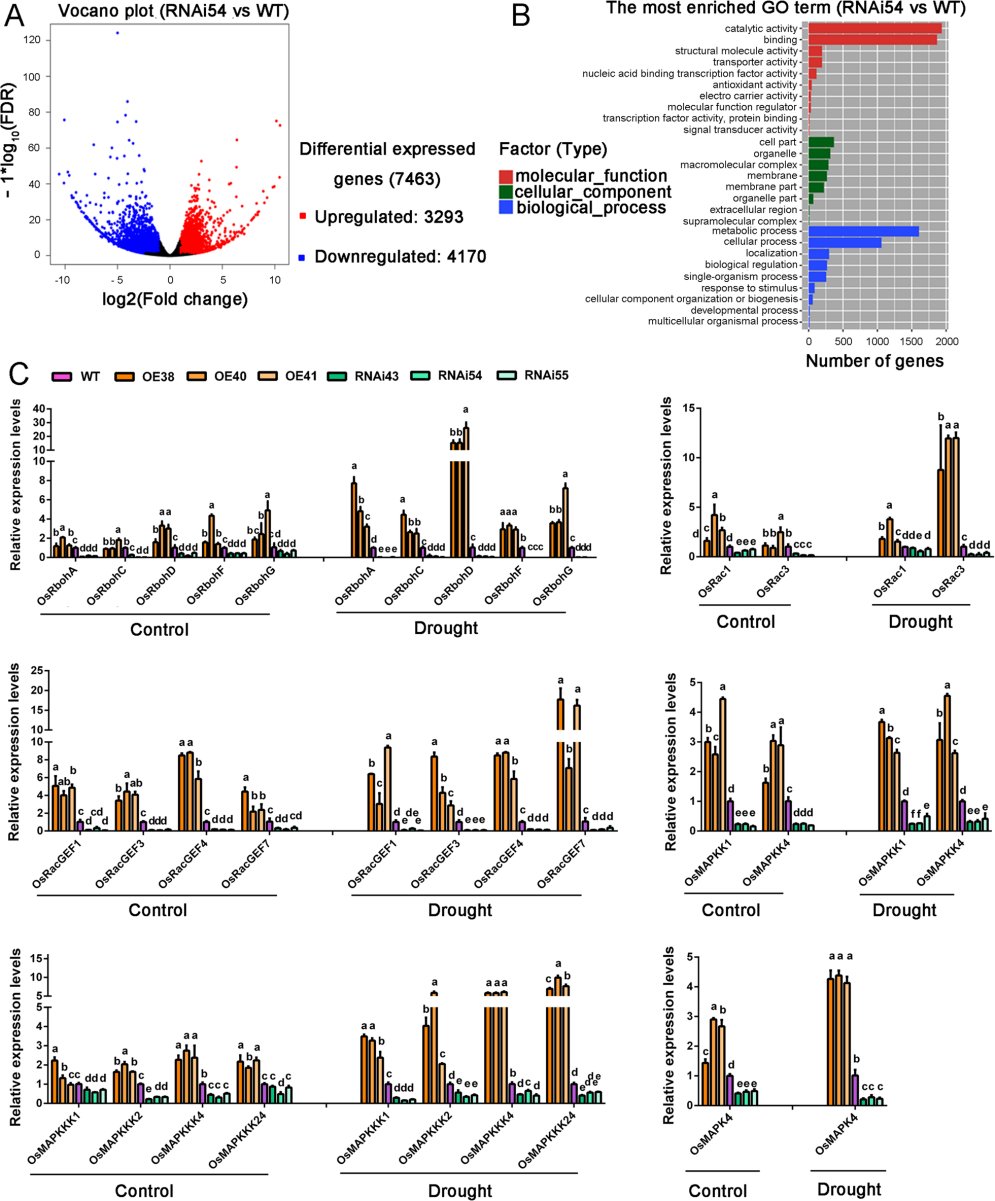
Transcription profiles of the differentially expressed genes between the *OsCBM1*-transgenic plants and the wild type (WT). **A** Comparison of expression patterns of differentially expressed genes identified between the WT (Nipponbare) and *OsCBM1*-RNAi transgenic plant RNAi54. The leaves of six-week-old plants of OsCBM1-RNAi54 and the WT grown in plastic pots filled with paddy soil were used for the transcriptional analysis. The red dots represent upregulated genes, whereas the blue dots represent downregulated genes in the RNAi54 plants as compared to that of the WT. **B** Significantly enriched GO categories of the differentially expressed genes. Results are summarized in three categories: biological process, cellular component, and molecular function. **C** Transcriptional expression profiles of several protein family genes under both normal growth and drought stress conditions. The transcripts of the selected genes were analyzed by qRT-PCR with *OsActin1* as the internal control. Error bars indicate SD from three replicates and the bars annotated with different letters represent values that are significantly different (*p* ≤ 0.05) according to a one-way ANOVA

To further elucidate the functions of OsCBM1 in plant development and stress tolerance, we performed an RNA-seq experiment to identify the differentially expressed genes (DEG) when *OsCBM1* was knocked down in rice. A total of 7463 genes were differentially expressed in the *OsCBM1*-downexpressing RNAi54 plants (Additional file 5: Table S3). Among these genes, 3293 were upregulated and 4170 were downregulated in the RNAi54 plants compared with that of the WT (Fig. 6A). GO analysis indicated that most enriched GO terms of DEG were associated with catalytic activity, cell part, and metabolic process (Fig. 6B). As shown in Additional file 6: Table S2, 40 genes, which were assigned to ATPases or GTPases, were differentially expressed in the RNAi54 plants as compared that of the WT. And 56 genes assigned to a pentatricopeptide repeat (PPR) domain-containing proteins and 15 genes encoding a set of ankyrin repeat (ANK) domain-containing proteins were also highly differentially expressed in the transgenic plants. Furthermore, a set of genes (13) encoding the Rapid ALkalinization Factors (RALFs) was also highly differentially expressed in the RNAi54 plants compared to that of the WT (Additional file 6: Table S2). Additionally, more than 170 differentially expressed genes were assigned to “Protein Kinases”, including 24 lectin-like protein kinases, 12 wall-associated receptor kinases, six stress-activated protein kinases, and 129 receptor-like protein kinases (Additional file 6: Table S2).

More interestingly, several sets of genes that encode respiratory burst oxidase homologs (Rbohs), Rop/Rac associated proteins (Racs), GEFs, mitogen-activated protein kinase (MAPK) cascade-related proteins, and drought-stress associated proteins were highly downregulated in the RNAi54 plants compared with that of the WT (Table 1). To further elucidate this issue, we analyzed the transcriptional expression profiles of OsRbohs, OsRacGEFs, OsRacs, and OsMAPK cascade family genes in both the OsCBM1-overexpressing, OsCBM1-RNAi transgenic and WT plants by qRT-PCR. As shown in Additional file 7: Fig. S5, almost all of the *OsRboh* genes are upregulated in the OE plants but downregulated in the RNAi plants, this was especially true for *OsRbohA*, *C*, *F,* and *I*, which showed very high coexpressions with *OsCBM1*. Similar results were observed for the *OsRacGEF* family genes. Among them, *OsRacGEF1*, *3, 4*, *6*, *8,* and *11* exhibited strong coordination in expression with *OsCBM1* in the transgenic plants. In the *OsRac* family genes, *OsRac1*, *3*, *6,* and *7* somewhat showed coexpression with *OsCBM1* because they were all significantly downregulated in the RNAi plant lines. Interestingly, a set of MAPK cascade-related protein genes were also highly exhibited in coexpression profiles with *OsCBM1*. Several MAPKKKs, namely *OsMAPKKK1*, *2*, *4*, potentially *24*, two MAPKKs, *OsMAPKK1* and *4*, and one MAPK, *OsMAPK4*, showed very strong coordination with the expression of *OsCBM1* (Additional file 7: Fig. S5).

**Table 1:** Selected genes that differentially expressed in the RNAi54 transgenic plants as compared to WT

RAP_Locus	MSU_Locus	Gene description	Fold change	*p*-value	Regulation
*Rice respiratory burst oxidase homologs*					
Os08g0453766	LOC_Os08g35210	Rice respiratory burst oxidase homolog, OsrbohE; Osrboh6	7.92	1.55E−08	Down
Os09g0438000	LOC_Os09g26660	Rice respiratory burst oxidase homolog, OsrbohB; Osrboh7	5.16	8.83E−10	Down
Os01g0734200	LOC_Os01g53294	Rice respiratory burst oxidase homolog, OsrbohA; Osrboh2	4.07	1.41E−06	Down
Os01g0360200	LOC_Os01g25820	Rice respiratory burst oxidase homolog, Osrboh1	2.59	7.01E−05	Down
*Rop/Rac associated proteins*					
Os02g0719000	LOC_Os02g48730	Rho GDP-dissociation inhibitor 1, putative, expressed	30.90	5.82E−08	Down
Os01g0917700	LOC_Os01g68890	P21-Rho-binding domain containing protein, putative, expressed	16.65	1.19E−11	Down
Os04g0627400	LOC_Os04g53580	P21-Rho-binding domain containing protein, putative, expressed	8.38	2.24E−03	Down
Os03g0847900	LOC_Os03g63060	P21-Rho-binding domain containing protein, putative, expressed	6.31	3.23E−12	Down
Os06g0318300	LOC_Os06g21340	Rho GDP-dissociation inhibitor 1, putative, expressed	2.35	1.39E−05	Down
Os04g0561200	LOC_Os04g47330	Rho-GTPase-activating protein-related, putative, expressed	11.43	2.53E−04	Ups
Os01g0757600	LOC_Os01g55280	OsRac5 partner; The myosin heavy chain-coding gene	2.45	2.25E−04	Down
*Guanine nucleotide exchange factors*					
Os04g0559100	LOC_Os04g47170	ATROPGEF7/ROPGEF7, putative, expressed	5.16	8.51E−06	Down
Os10g0550300	LOC_Os10g40270	ATROPGEF7/ROPGEF7, putative, expressed	5.02	1.67E−02	Down
Os02g0702600	LOC_Os02g47420	ATROPGEF7/ROPGEF7, putative, expressed	4.85	3.31E−04	Down
Os5g0454200	LOC_Os05g38000	Guanine nucleotide exchange factor, OsRopGEF10	4.64	1.34E−02	Down
*Mitogen-activated protein kinases*					
Os01g0629900	LOC_Os01g43910	Mitogen-activated protein kinase, OsMAPK20-1	3.84	1.99E−08	Down
Os06g0699400	LOC_Os06g48590	Mitogen-activated protein kinase, OsMAPK4	2.08	9.20E−05	Down
Os05g0566400	LOC_Os05g49140	Mitogen-activated protein kinase, OsMAPK7	2.01	2.08E−03	Down
Os06g0724900	LOC_Os06g50920	Mitogen-activated protein kinase kinase kinase, ILA1	3.05	2.30E−05	Ups
Os02g0135200	LOC_Os02g04230	Mitogen-activated protein kinase, OsMAPK13	3.69	1.90E−08	Ups
*Drought-stress associated proteins*					
Os02g0669100	LOC_Os02g44870	Dehydration-stress inducible protein 1	2.14	1.15E−04	Down
Os07g0569700	LOC_Os07g38240	C2H2 transcription factor, stress associated protein 16, OsSAP16	2.05	3.50E−04	Down
Os03g0793000	LOC_Os03g57900	Stress associated protein 7, OsSAP7	2.03	8.74E−04	Down
Os01g0233000	LOC_Os01g13210	Salt stress root protein RS1, putative, expressed	2.79	5.19E−04	Down
Os03g0179400	LOC_Os03g08170	Drought-inducible receptor-like cytoplasmic kinase, OsRLCK103	3.25	1.70E−07	Down
Os03g0286900	LOC_Os03g17790	Drought resistance, rare cold-inducible 2–5, OsRCI2-5	2.82	5.17E−03	Down

The expression levels of the genes were detected by RNA-seq experiment

Next, we selected some members of these gene families to verify their coexpression relationship with *OsCBM1* when the plants were subjected to drought stress conditions. As seen in Fig. 6C, all the selected genes from *OsRboh*, *OsRac*, *OsRacGEF,* and *OsMAPK* cascade families showed strong coexpression patterns with *OsCBM1* when it was differentially expressed in transgenic plants both under normal growth and drought stress conditions. Almost all of the selected genes from these protein families were substantially upregulated in the OE plants but significantly downregulated in the RNAi plants as compared to that of the WT. Additionally, the transcripts of most of the selected genes, including *OsRbohA/C/D/G*, *OsRac3*, *OsRacGEF1/7*, *OsMAPKKK1/2/4/24,* and *OsMAPK4,* were markedly increased by drought stress in the OE plants (Fig. 6C).

## Discussion

Malectin and malectin-like domain-containing proteins belong to a large protein family that plays important roles in plant growth regulation, cell-wall integrity, signaling transduction, immunity, and abiotic stress response (Nissen et al. 2016; Franck et al. 2018; Jing et al. 2020). The first malectin protein identified was located in the ER and functioned in protein N-glycosylation in animal cells (Schallus et al. 2008). In plants, a small plant-specific putative glycosylphosphatidylinositol (GPI)-anchored protein LORELEI (LRE) was localized to the filiform apparatus together with the malectin-like receptor kinase FER and chaperones FER on the transport way from ER to the filiform apparatus (Liu et al. 2016). LRE, and its closest relative LORELEI-like-GPI-AP1 (LLG1), also acted as coreceptors of FER in its shuttling from the ER to the cell surface (Li et al. 2015). Moreover, it was found that both RHO-GTPASE OF PLANTS 2 (ROP2) and RALF1 directly interacted with FER, LRE, and LLG1, implying that LRE and LLG1 acted as chaperones and possibly coreceptors for FER during plant immunity (Franck et al. 2018). LLG1 was also required for flg22-induced BOTRYTIS-INDUCED KINASE 1 (BIK1) phosphorylation and ROS production (Stegmann et al. 2017). However, whether FER and other malectin and malectin-like domain-containing proteins directly interact with the NADPH oxidases (Rbohs) to regulate ROS production is still under investigation. In an our recent study, a total of 84 malectin/malectin-like domain-containing protein genes, including 67 *MRLKs* and 17 *MRLPs* were identified in the rice genome (Jing et al. 2020), but their roles and functional properties in the plant development and stress tolerance were largely unknown. In the present study, we found that one carbohydrate-binding malectin-like domain-containing protein OsCBM1 played an important role in drought tolerance of rice. It can interact with OsRbohA and OsRacGEF1 contributing to ROS production. The results provided herein demonstrate a new functional mechanism for the malectin-like domain-containing proteins in rice stress tolerance.

### OsCBM1 is Both an ER and PM Located Malectin-Like Domain-Containing Protein Functioning in Development and Drought Stress Tolerance of Rice

As described above, CBMs in plants are usually assigned to the CrRLK1L family proteins because the most well-characterized CrRLK1L contains a malectin-like domain and a kinase domain in structure (Boisson-Dernier et al. 2011; Hok et al. 2011; Lindner et al. 2012; Galindo-Trigo et al. 2016; Yeh et al. 2016; Bellande et al. 2017; Hu et al. 2018). These malectin-like receptor kinases participate in many important biological processes from cell-wall integrity to respond to numerous biotic and abiotic stresses, as reviewed by Franck et al. (2018). However, studies have shown that some CBMs have only malectin and/or malectin-like domains in structure and no kinase domain was identified (Zhang et al. 2016; Bellande et al. 2017; Jing et al. 2020). The biological roles of this kind of CBMs in plants have not been examined. Herein, we showed that OsCBM1, which has only a malectin-like domain-containing protein, is located at both the ER and PM (Fig. 1A, C). Its transcripts were dominantly expressed in leaves of rice and could be greatly stimulated by many hormonal treatments and environmental stresses (Fig. 1B, D), demonstrating that OsCBM1 is a stress-responsive CBM protein.

Additionally, knockdown of *OsCBM1* significantly reduced plant tillering and drought tolerance but increased the plant height, panicle weight, and seed setting, whereas overexpression of the protein enhanced the plant tolerance to drought (Figs. 2, 3A, B), suggesting that this protein positively contributes drought tolerance but negatively influences seed yield in rice. The stomatal aperture differed in the different types of transgenic plants and this might be a major reason for the drought tolerance and yield differential between the transgenic plant lines and that of the WT (Fig. 3C, D). The fact that the *OsCBM1*-RNAi transgenic lines possess greater numbers of stomata and a higher percentage of opening ones implied a higher water loss rate for this kind of plant during development, which would benefit nutrient and substrate transport, and therefore, providing an advantage for growth and yield production of the plants under well-watered growth conditions. On the other hand, the *OsCBM1*-RNAi possessed greater numbers of stomata with a higher percentage of openings and conductance of stomata, which would reinforce water loss such that drought tolerance was reduced for these plants under water deficit conditions (Fig. 3C, D, Additional file 2: Fig. S2). Together, these results suggest that OsCBM1 is a novel malectin-like domain-containing protein that functions in plant development and stress tolerance, particularly drought.

### A GEF/RAC-Dependent Activation of OsRbohA Could Participates in the OsCBM1-Mediated ROS Production for Drought Tolerance

ROS production plays a pivotal role in the induction of robust immune responses and stress tolerance of plants. Previous studies have shown that many RLKs, including the malectin-like domain-containing CrRLK1L family proteins FER and ANX1/2, may trigger ROS production by regulating the PM NADPH oxidases (Rbohs) during plant immunity and normal growth regulation (Kawasaki et al. 2017; Franck et al. 2018). It was suggested that FER interacts with the GPI-anchored proteins EARLY NODULIN 14 (EN14) and LRE; the latter putatively promotes the function of a yet unknown downstream NADPH oxidase protein triggering ROS accumulation in the female gametophytes of plants (Franck et al. 2018). The two other CrRLK1L proteins, ANX1 and ANX2, act upstream of the ROS-producing NADPH oxidases RbohH and RbohJ to regulate pollen cell-wall integrity (Boisson-Dernier et al. 2013). Additionally, all the FER and ANX1/2 function in plant immunity. FER positively influenced PAMP-triggered immunity (Stegmann et al. 2017); whereas ANX1/2 appeared to negatively regulate both PAMP-triggered immunity and effector-triggered immunity (Mang et al. 2017). To date, among the 10 ROS-producing NADPH oxidases in *Arabidopsis thaliana* (Baxter et al. 2014), RbohC, RbohD, RbohF, RbohH, and RbohJ have been implicated in CrRLK1L-mediated pathways. In the present study, OsCBM1 positively mediates ROS production, and OsRbohA is involved in the process (Figs. 3, 4). OsRbohA is a key ROS producer in rice and participates in drought tolerance (Wang et al. 2016). Additionally, besides OsRbohA, other OsRbohs, including OsRbohC, D, F, and G, might also participate in OsCBM1-mediated ROS production because their transcriptional expression profiles exhibited strong coordination with *OsCBM1* and their transcripts could be greatly stimulated by drought in the *OsCBM1*-overexpressing plants (Additional file 7: Fig. S5 and Fig. 6C). In addition, LCI, BiFC and pull-down assay showed that OsCBM1 directly interacted with OsRbohA (Fig. 4C–E). These results indicated that OsCBM1 positively contributes ROS production most likely by activating OsRbohA, or other OsRbohs in rice.

The function of OsCBM1 in activation of the rice NADPH oxidases for ROS production might be involved in its interaction with OsRacGEF1. OsRacGEF1 belongs to a plant-specific ROP nucleotide exchanger (PRONE)-type GDP/GTP exchange factor (GEF) family and it was identified as the specific GEF for OsRac1. As a rice Rac/Rop GTPase, OsRac1 plays important roles in plant immune responses (Ono et al. 2001; Kawasaki et al. 2006; Wong et al. 2007; Akamatsu et al. 2013) and positively regulates ROS production via interaction with the N-terminal region of OsRbohs (Wong et al. 2007; Nagano et al. 2016). OsRacGEF1 is an ER-located protein but it may interact with the cytoplasmic domain of the PM RLK protein and OsCERK1 may be transported from the ER to PM through a vesicle trafficking pathway and be phosphorylated by OsCERK1 to activate OsRac1 during chitin-induced immunity (Akamatsu et al. 2013). In *Arabidopsis*, it was reported that RopGEF1, RopGEF7, RopGEF10, RopGEF14, and Rop2 directly interact with the malectin-like receptor kinase FER kinase domain (Duan et al. 2010). Rop2 further interacts with the N-terminal part of RbohD (Li et al. 2015). Additionally, FER also interacts with Rop11/ARac10 and negatively regulates ABA signaling via ABI2 (Yu et al. 2012). Lastly, the activated Rac/Rops target the ROS-producing plant NADPH oxidases (Carol et al. 2005; Wong et al. 2007), to regulate ROS production. Herein, OsCBM1 directly interacted with OsRacGEF1 (Fig. 5), and both proteins strongly co-localized in subcellular compartments, including ER and PM (Additional file 3: Fig. S3). OsRacGEF1 could be transported from the ER to PM to activate OsRac1 (Akamatsu et al. 2013). In this study, the PM localization of OsRacGEF1 seemed to be diminished in the *OsCBM1*-RNAi54 plants (Additional file 4: Fig. S4), implying that the lack of OsCBM1 might affect the transport of OsRacGEF1 from ER to PM. These results suggested that OsCBM1 might undergo a similar transport mechanism as OsRacGEF1 does in OsCERK1-mediated signaling (Akamatsu et al. 2013) during rice drought stress response. OsCBM1 may act as a chaperone of OsRacGEF1 for the protein translocation from ER to PM.

It should be noticed that besides OsRacGEF1 directly interacting with OsCBM1 and exhibiting strong coordination in transcriptional expression with *OsCBM1* in the transgenic plants (Figs. 5, 6; Additional file 7: Fig. S5), three other RacGEF genes, including *OsRacGEF3*, *4,* and *7*, also showed strong coexpression with *OsCBM1* both under normal growth and drought stress conditions (Fig. 6C), implying these OsRacGEFs might also play a role in OsCBM1-mediated ROS production even though the molecular interactions between OsCBM1 and these OsRacGEFs have not been fully verified yet. Besides *OsRac1*, other *OsRac* genes including *OsRac2*/*3*, and possibly *OsRac5/6*, also exhibited strong coordination with the expression of *OsCBM1*, further suggesting that the role of OsCBM1 in ROS production might be involved in GEF/RAC-dependent activation of NADPH oxidases. The interaction between OsRac GTPases and OsRbohs is ubiquitous and besides OsRac1, other Racs, including OsRac2 and OsRac7, interact with OsRbohA, B, C, and D (Wong et al. 2007). These results demonstrated that a GEF/RAC-dependent activation of Rbohs might be involved in OsCBM1-mediated drought tolerance by positively regulating ROS production in rice. Furthermore, the expression levels of *OsRbohA* and several other members, such as OsRbohC/D/F/G, were substantially upregulated in the *OsCBM1*-OE plants but downregulated in the RNAi plants under both normal growth and drought stress conditions (Fig. 6). Considering that *OsRbohA* and *OsCBM1* exhibited a very strong coexpression relationship, in which the expression levels of *OsCBM1* were significantly downregulated in the *osrbohA* mutant (Fig. 4A, B), the results obtained herein suggest that a GEF/RAC-dependent activation of OsRbohA and/or other OsRbohs might participate in the OsCBM1-triggered ROS production for drought tolerance.

### The Change of *CBM1* Transcriptional Level Might Affect Multiple Signaling Pathways

Based on the results from the RNA-seq assay, a huge number of genes were found to be differentially expressed when OsCBM1 was downregulated in rice (Fig. 6A, B; Additional file 5: Table S3). Among those genes, 40 genes were assigned to ATPases or GTPases with some members substantially downregulated in the RNAi54 plants, such as the genes encoding ATPase 2 and ATPase 3, and upregulated in the transgenic plants, such as the gene encoding a Rho-GTPase activating protein (Os04g47330). ATPases and GTPases are central actors in bioenergetics, signaling, and many other cellular processes (Leipe et al. 2002; Wendler et al. 2012), implying a crucial role of OsCBM1 in plant basic metabolic processes, such as bioenergetics and signaling although we do not know much about the details at this stage. RALFs are small cysteine-rich peptides known to be involved in various aspects of plant development and growth (Campbell and Turner 2017). They may act as ligands for the malectin-like receptor kinases and many of them have been identified as participating in the malectin-like receptor kinases, such as FER and ANX1/2, associated with signaling during immunity, pollen tube growth, root and hypocotyl elongation, and salt stress tolerance (Ge et al. 2017; Mecchia et al. 2017; Franck et al. 2018; Zhao et al. 2018). A set of RALFs genes (13) were differentially expressed in the *OsCBM1*-RNAi54 plants with most of them (11) substantially downregulated in the transgenic plants (Additional file 6: Table S2), suggesting that these RALFs could participate in the OsCBM1-mediated processes.

Additionally, OsCBM1 might also be involved in modular RNA and protein activity in plants. PPR repeat domain proteins are a large family of modular RNA-binding proteins mediating several aspects of gene expression primarily in organelles but also the nucleus (Manna 2015). In *Arabidopsis*, a PPR protein THA8L was found to be required for the splicing of specific genes involved in the biogenesis of chloroplast thylakoid membranes (Ban et al. 2013). In the present study, a total of 56 genes encoding PPR repeat domain-containing proteins were differentially expressed in the *OsCBM1*-RNAi54 plants, where 55 were upregulated (Additional file 2: Table S2), indicating that OsCBM1 was an ER-located malectin-like domain-containing protein, which might also function in the RNA splicing or editing process in rice. Another large protein family is the ANK repeat domain-containing proteins, which function in protein–protein interactions and have been found in numerous proteins with diverse functions (Michaely and Bennett 1992; Bork 1993; Mosavi et al. 2004). In animals, some ANK proteins, for example, Ankrd27, participate in membrane fusion events, such as functioning as a guanine exchange factor (GEF) for Rab proteins (https://www.uniprot.org/uniprot/Q3UMR0). In plants, some ANK genes are tissue-specifically expressed and their transcripts could be stimulated by a number of phytohormonal and abiotic stress treatments, suggesting their roles in plant growth and development, as well as in stress responses (Yuan et al. 2013). Herein, 15 ANK genes were altered in expression in the RNAi54 plants compared with that of the WT, suggesting that these ANKs could also be involved in the OsCBM1-mediated process in rice. Because OsCBM1 directly interacts with OsRacGEF1, whether these ANKs function in the interactions of OsCBM1 with RacGEF proteins requires further study. More interestingly, more than 170 RLK genes were differentially expressed with a substantial number being downregulated when the expression of *OsCBM1* was knocked down (Additional file 2: Table S2). Although we do not know the exact meaning at this stage, the transcriptional expression levels of many RLK genes were altered by the *OsCBM1*-knocked down, demonstrating the vital role of OsCBM1 in plant signaling because most of RLKs function in signaling reception and transduction in plants (Tena et al. 2011; Couto and Zipfel 2016; Zhou and Yang 2016; Yu et al. 2017).

Interestingly, a set of genes encoding MAPK cascade-related proteins and drought-stress associated proteins also exhibited strong coexpression with *OsCBM1* in the transgenic plants (Table 1; Fig. 6; Additional file 7: Fig. S5), suggesting that a complicated regulatory module might be involved in OsCBM1-mediated ROS production and drought stress response. In plant immunity, many studies have shown that the MAPK cascade plays key roles in RLK signaling transduction and multiple defense responses (Kishi-Kaboshi et al. 2010; Meng and Zhang, 2013; Kawasaki et al. 2017; Yamada et al. 2017). The Rbohs-dependent ROS production usually acts as an upstream regulator for MAPK cascade signaling (Kawasaki et al. 2017; Yamada et al. 2017; Zhang et al. 2018). Herein, both the RNA-seq results (Table 1) and qRT-PCR analysis (Fig. 6C; Additional file 7: Fig. S5) indicated that many MAPK cascade signaling proteins, as well as a set of genes related to drought stress tolerance function in OsCBM1-mediated ROS production and stress tolerance. Previous studies have shown that MAPK activation is independent of ROS production in *Arabidopsis* (Xu et al. 2014). A chemical genetic approach demonstrated that MPK3/MPK6 activation and NADPH oxidase-mediated oxidative burst are two independent signaling events in plant immunity (Xu et al. 2014). However, the results obtained here showed that an OsMAPKKK1/2/4/24-OsMAPKK1/4-OsMAPK4 signaling cascade might be involved in the OsCBM1-mediated process. Apparently, this process is related to Rbohs-mediated ROS production since OsCBM1 directly interacts with OsRbohA. Whether or not the MAPK cascade activation and Rbohs-mediated ROS production are two dependent signaling in drought tolerance, requires further study.

**Fig. 7:**
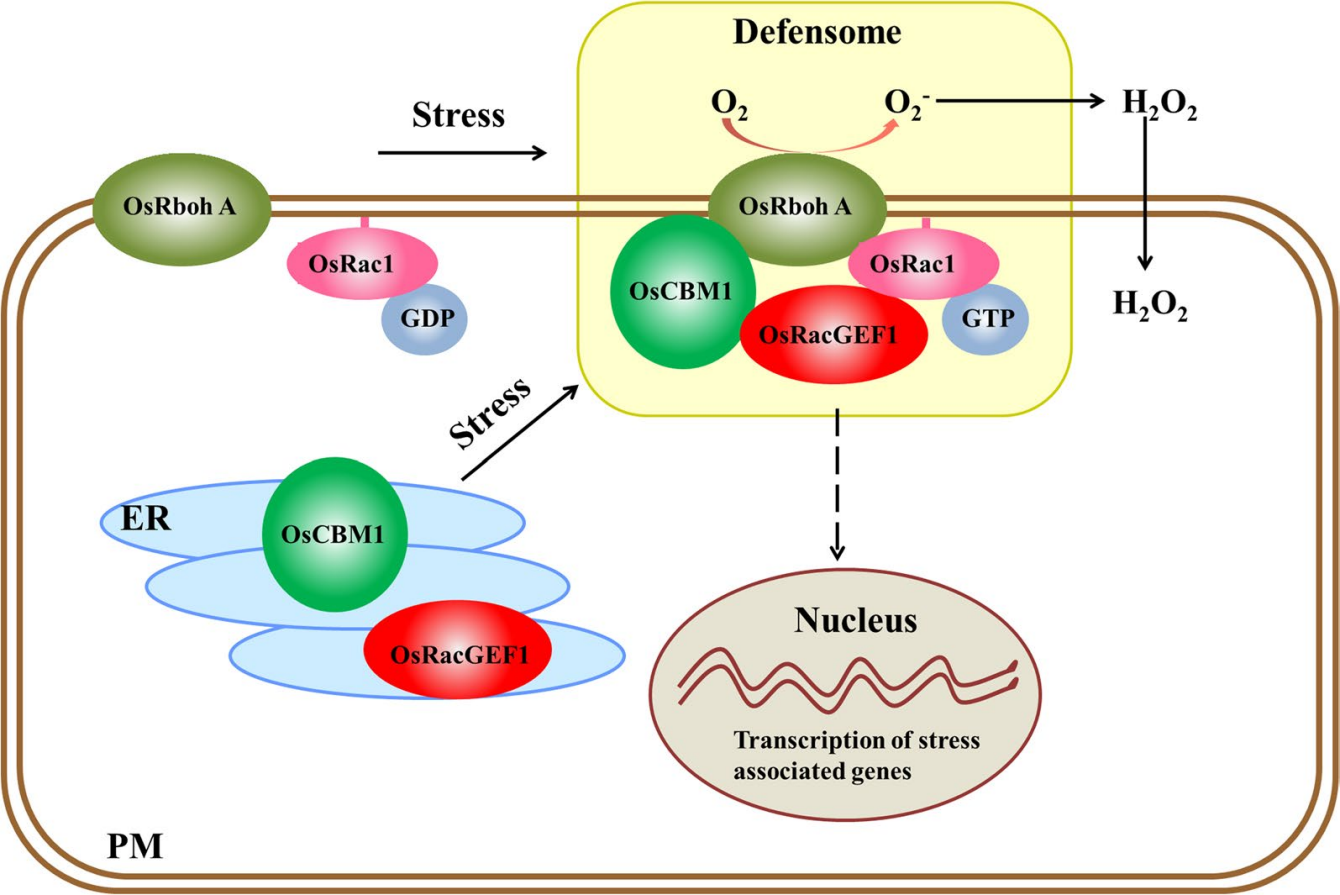
Working model for OsCBM1 participating in drought-stress tolerance by regulating NADPH oxidase-mediated ROS production. OsCBM1 and OsRacGEF1 are co-located at the ER. When the rice plants suffered from drought stress, OsCBM1 can be transported from the ER to the PM together with OsRacGEF1, where they formed a defensome complex with OsRbohA, and then OsRbohA was activated. The activated OsRbohA enhanced ROS production in apoplastic space, which contributed to drought-stress tolerance of the plants possibly by regulating a set of genes expression

Taken together, this study demonstrated that an only malectin-like domain-containing protein, OsCBM1, which is localized both in ER and PM, can be greatly stimulated by a number of environmental factors. OsCBM1 interacts with OsRacGEF1 and might be transported to the PM together with OsRacGEF1, where they formed a defensome complex with OsRbohA and OsRac1 when the plants suffered from drought stress, and then, OsRbohA was activated. Activated OsRbohA mediates ROS production and contributes to drought-stress tolerance (Fig. 7). The OsCBM1-mediated drought tolerance of rice might involve multiple signaling pathways, but the details require further study.

## Materials and Methods

### Plant Materials and Growth Conditions

*Oryza sativa* subsp*. japonica* Nipponbare was used as the WT plant in all the experiments. The experiments were performed in the State Key Laboratory of Crops Stress Biology for Arid Areas, Northwest A&F University, Yangling, China. Rice seeds were sterilized with 0.5% (w/v) sodium hypochlorite (NaClO) for 4 h and then germinated on humid cheesecloth in darkness. The well-germinated seeds were selected and grown in a growth chamber with the application of Hoagland solution (Hoagland and Arnon 1950). Healthy and uniform seedlings were selected for further analysis or treatments, or transferred to plastic pots (soil) in a greenhouse with each pot filled by same weight soil from paddy fields.

*Nicotiana benthamiana* was also used in this experiment. Seeds of *N. benthamiana* were germinated on sterile 1/2 MS solid medium at 28 °C for 7 d and then grown in plastic pots filled with a substrate (Growing Media Europe AISBL, Belgium) in a growth chamber. Four-week-old seedlings were used for the following experiments, including the analysis of protein subcellular localization, visualization of ROS production, assays of the firefly LCI, and immunoprecipitation (Co-IP). The details for plant materials and growth conditions are presented in Additional file 9: Supporting experimental procedures Methods S1.

### Expression Profile Analysis

The expression profiles of *OsCBM1* in different plant organs or tissues and under different environmental conditions were analyzed as previously described (Wang et al. 2016). For the tissue-specific expression analysis, the WT of rice plants were allowed to grow in paddy fields and the different plant organs or tissues were collected at various developmental stages. After harvest, the samples were immediately frozen in liquid nitrogen and stored at − 80 °C until further analyses. To identify the inducible expression profiles of *OsCBM1*, the young seedlings (2-week-old) of WT plants grown in the growth chamber, as described above, were exposed to various abiotic stresses and phytohormones. The treatments were conducted as previously described (Muhammad et al. 2018). The expression levels of the genes were detected by qRT-PCR with *OsActin1* (Gene ID: KC140126) used as an internal control. All the RT-PCR and qRT-PCR primers used in this study are listed in Additional file 8: Table S1 and the details for the experiments are presented in Additional file 9: Supporting experimental procedures Methods S1.

### Subcellular Location Analysis

Subcellular localization of OsCBM1 and the co-localization of OsCBM1 with OsRacGEF1 in subcellular compartments were determined using an *N. benthamiana* transient transformation system (Chen et al. 2009). AtCBL1n-mCherry served as a marker for PM protein localization (Batistic et al. 2010) and AtCHS-mCherry was used as a marker for ER protein localization (Dana et al. 2006). For the analysis of OsRacGEF1 localization in the *OsCBM1*-RNAi transgenic plants, the *OsRacGEF1*-*mCherry* containing vector was transformed into rice protoplasts from 7–10 d old WT and *OsCBM1*-RNAi54 seedlings. The fluorescence was observed under a confocal microscope (A1R, Nikon, Tokyo, Japan). The primers used for the generation of the plasmids are listed in Additional file 8: Table S1 and the experimental details are presented in Additional file 9: Supporting experimental procedures Methods S1.

### Generation of Transgenic Plants and Their Phenotypes

To construct the *OsCBM1*-OE transgenic lines, the ORF sequence of *OsCBM1* was amplified and then inserted into the binary vector pCAMBIA1301 to generate an overexpressing plasmid under the control of the CaMV 35S promoter. To construct the *OsCBM1*-RNA interference (RNAi) transgenic lines, the partial ORF sequence of *OsCBM1* was amplified and then connected by both the forward and reverse sequences to the two ends of the intron on the vector pTCK303. The constructs were then introduced into *A. tumefaciens* strain EHA105 and used to infect the callus of WT Nipponbare rice. Transgenic rice plants were generated as previously described (Toki et al. 2006). The expression levels of *OsCBM1* in the transgenic lines were detected by real-time qRT-PCR with the WT as the control. The primers used for the generation of the plasmids for transgenic plants are listed in Additional file 8: Table S1. Agronomic traits of the OE, RNAi, and WT were measured using 30 plants grown in the field at the ripening stage for each type of plant.

### Analysis of Stomata Aperture

At least 1000 stomata taken from six different leaves were photographed with a scanning electron microscope (SU8010, Hitachi, Japan) to study the stomata aperture in the OE, RNAi, and WT plants. Strips of abaxial epidermis of the rice leaves were used for the analysis. The status of 100 stomata was analyzed in ImageJ software by measuring the width and length of stomata in the leaves for each type of plant (Zhang et al. 2011). The details for the experiment are presented in Additional file 9: Supporting experimental procedures Methods S1.

### Drought Treatment and Determination of Related Parameters

The well-germinated seeds of the OE, RNAi, and WT plants, as described above, were sown in plastic pots filled with Sunshine MVP potting soil (Yangling, China). Six-week-old plants were used for drought treatment by withholding water. The last fully expanded leaves under both normal growth and drought stress conditions were collected for the determination of ROS production, photosynthetic characteristics, and gene expression profiles. A Li-6400 portable photosynthesis system (Li-COR Biosciences, Lincoln, NE, USA) was used to measure the photosynthetic characteristics as described by Wu et al. (2011). The transcription profiles of differentially expressed genes between the OsCBM1-transgenic plants and WT under drought were analyzed with qRT-PCR, as described above. For the assay of drought tolerance between the OE, RNAi, and WT plants, the 4-week-old seedlings grown in Sunshine MVP potting soils were directly treated by withholding water and the survival rates of the plants were recorded after watering. The details for the experiments are presented in Additional file 9: Supporting experimental procedures Methods S1.

### Detection of ROS Production

Histochemical analysis of ROS (H_2_O_2_ and O_2_^–^) production in leaves of the OE, RNAi, and WT plants were conducted as previously described (Wang et al. 2016). The detached leaves from 6-week-old plants grown under normal growth conditions were used for the histochemistry assay. Contents of H_2_O_2_ in leaves of different types of rice plants were determined with a Hydrogen Peroxide Assay Kit (Solarbio Institute of Biotechnology, Beijing, China) by following the manufacturer’s instructions. The O_2_^–^ production rate was measured as described by Jiang and Zhang (2003) by monitoring the reduction of sodium 3′-[1-[phenylamino-carbonyl]-3, 4-tetrazolium]-bis (4-methoxy-6-nitro) benzenesulfonic acid hydrate (XTT) in the presence of O_2_^–^. ROS production in protoplasts was visualized via fluorescence of 2, 7-dichlorodihydrofluorescein dilacerate acetyl ester (H_2_DCFDA) as previously described (Wang et al. 2016). The fluorescence visualization and intensity in root tips were detected using a scanning confocal microscope (Leica TCS-SP2, excitation 488 nm, emission 525 nm). Visualization of ROS in situ in *N. benthamiana* leaves was performed as described by Wong et al. (2007) with *A. tumefaciens* strain GV3101 harboring the plasmid pCAMBIA1300-221-cMyc vector carrying the ORF of *OsCBM1*, *OsRacGEF1.* These together with an empty vector were used to infiltrate *N. benthamiana* leaves. The DAB-stained leaves were scanned and pixel intensity of agroinfiltrated regions was quantified by the ImageJ (National Institutes of Health) software. The details for these experiments are presented in Additional file 9: Supporting experimental procedures Methods S1.

### Pull-Down Assay

The protein expression in vitro and purification were conducted as described by Chen et al. (2009). In the interaction analysis of OsRacGEF1 and OsCBM1, the full-length coding sequence of *OsRacGEF1* was amplified and ligated into the pMAL-c2X vector for the preparation of the recombinant MBP-OsRacGEF1 protein and the coding sequence of *OsCBM1* was amplified and ligated into the pET-32a vector for the preparation of the recombinant His-OsCBM1 protein (Finkelstein and Lynch 2000; Nakamura et al. 2001). The pull-down assay of OsCBM1 with OsRacGEF1 was performed according to the method described by Zhang et al. (2010). In the interaction analysis of OsRbohA and OsCBM1, the NADPH-Ox domain at the N-terminal of *OsRbohA* (coding 360 amino acid residues at the N-terminal) was amplified and ligated into the pMAL-c2X vector for the preparation of the recombinant MBP-OsRbohA-ND protein, and the coding sequence of *OsCBM1* was amplified and ligated into the pET-32a vector for the preparation of the recombinant His-OsCBM1 protein. The primers used for the generation of the recombinant proteins are listed in Additional file 8: Table S1 and the details for the experiment are presented in Additional file 9: Supporting experimental procedures Methods S1.

### Split-Ubiquitin Yeast Two-Hybrid Assays

The full-length coding sequences of *OsRacGEF1* and *OsCBM1* were amplified and cloned into vectors pBT-STE and pPR-SUC, respectively. The plasmids were co-transformed into *Saccharomyces cerevisiae* NMY51 yeast cells and the performance of protein interaction analysis was conducted according to the Yeast Protocols Handbook (Clontech, Mountain View, CA, USA). The primers used for generating the various vectors are listed in Additional file 8: Table S1 and the details for the experiment are presented in Additional file 9: Supporting experimental procedures Methods S1.

### Firefly LCI Assay

The LCI assays for the protein interaction detection were performed in *N. benthamiana* leaves. In the interaction analysis of OsRacGEF1 and OsCBM1, the full-length of coding regions of *OsRacGEF1* and *OsCBM1* were fused with the C- and N-terminal parts of the luciferase reporter gene LUC, respectively. In the interaction analysis of OsRbohA and OsCBM1, the full-length of coding regions of *OsRbohA* and *OsCBM1* were fused with the C- and N-terminal parts of the luciferase reporter gene LUC, respectively. LUC activity was detected with a living plant molecular marker imaging system (Lumazone Pylon 2048B, Princeton, American). The list of primer sets used for the generation of the constructs are listed in Additional file 8: Table S1 and the details for the experiment are presented in Additional file 9: Supporting experimental procedures Methods S1.

### Immunoprecipitation Assay

The full-length coding sequence of *OsRacGEF1* was amplified and ligated into the pCAMBIA 1300-221-cMyc vector for the preparation of recombinant cMyc-OsRacGEF1 protein. The full-length coding sequence of *OsCBM1* was fused upstream of the green fluorescent protein (GFP) gene and inserted into the binary vector pCAMBIA1301-eGFP for the preparation of recombinant OsCBM1-eGFP proteins. The transformation vectors were introduced into *A. tumefaciens strain* GV3101 and then used to transform the leaves of *N. benthamiana*. The do-immunoprecipitation (Co-IP) assay was conducted with previously described (Zhang et al. 2018). The primer sets used for the generation of the constructs are listed in Additional file 8: Table S1 and the details for the experiment are presented in Additional file 9: Supporting experimental procedures Methods S1.

### Bimolecular Fluorescence Complementation (BiFC) Assay

Coding sequences of *OsCBM1* and *OsRbohA* were amplified by PCR and cloned into the pSPYCE(MR) and pSPYNE(R)173 vectors, respectively (Hu et al. 2013). All of the cloning plasmids were introduced into *Agrobacterium* strain GV3101 and then coexpressed in *N. benthamiana* leaves as described previously (Hu et al. 2013). At 48 h after infiltration, the fluorescence signal of enhanced yellow fluorescent protein (eYFP) was observed.

### RNA-seq Analysis

For transcriptional analysis, the leaves of 6-week-old plants of *OaCBM1*-RNAi54 and WT (Nipponbare) plants grown in plastic pots filled with paddy soil were harvested and stored immediately in liquid nitrogen. The sequences were processed and analyzed by GENEWIZ, Inc., Suzhou, China. Only genes with associated transcript levels that had increased or decreased more than twofold and had an associated p-value of < 0.05 were used for further analysis. The details for the experiment are presented in Additional file 9: Supporting experimental procedures Methods S1.

### Statistical Analyses

Student’s *t*-tests were performed using SPSS 11.5 software (SPSS, Inc., Chicago, IL). Differences were considered significant at *p* ≤ 0.05 based on a one-way analysis of variance (ANOVA).

## Supplementary Information

**Additional file 1: Figure S1**. Expression levels of *OsCBM1* in the wild-type (WT), *OsCBM1*-overexpressing (OE), and RNA interference (RNAi) plants at tillering stages detected by semi-quantitative RT-PCR, using *OsActin1* as the internal control.

**Additional file 2: Figure S2**. Photosynthetic rate, stomatal conductance, transpiration rate, and water use efficiency in wild-type (WT), OsCBM1-overexpressing (OE), and RNA interference (RNAi) transgenic plants under both normal growth and drought stress conditions. Six-week-old plants grown in pots filled with equal amounts of paddy soil were subjected to drought stress treatment by withholding water. After 10 d, the last fully expanded leaves were used for the analysis. Data are means of three biological replicates (n = 12). Bars annotated with different letters represent values that are significantly different (*p* ≤ 0.05) according to a one-way ANOVA analysis.

**Additional file 3: Figure S3**. Co-localization analysis of OsCBM1 and OsRacGEF1. Subcellular localization of OsCBM1 and OsRacGEF1 were detected using the *Agrobacterium*-mediated transformation with both *N. benthamiana* epidermal cells and protoplasts, respectively. The plasmids containing *eGFP*, *mCherry*, *OsCBM1-eGFP*, or *OsRacGEF1-mCherry* genes were under the control of the CaMV 35S promoter. Bars = 10 μm. DIC, bright field.

**Additional file 4: Figure S4**. Subcellular localization of OsRacGEF1 in protoplasts of the OsCBM1-RNAi54 transgenic plants and wild type (WT). Subcellular localization of OsRacGEF1 was analyzed with a rice protoplast transient transformation system (Bars = 10 μm). The plasmid containing *mCherry* and *OsRacGEF1-mCherry* genes were under the control of the CaMV 35S promoter. DIC, bright field.

**Additional file 5: Table S3**. The original data of RNA-seq experiments.

**Additional file 6: Table S2**. Several gene groups that are differentially expressed in the OsCBM1-downexpressing RNAi54 plants as compared to WT.

**Additional file 7: Figure S5**. Transcriptional expression profiles of several protein family genes. Expression levels were detected by qRT-PCR with *OsActin1* used as the internal control. Error bars indicate SD from three biological replicates and the bars annotated with different letters represent values that are significantly different (*p* ≤ 0.05) according to a one-way ANOVA analysis.

**Additional file 8: Table S1**. The primer sequences used in the study.

**Additional file 9**. Supporting experimental procedures Methods S1.

## Data Availability

All data supporting the findings of this study are available within the paper and within its supplementary materials published online.

## Abbreviations

ABA: Abscisic acid
CBM: Carbohydrate-binding malectin/malectin-like domain-containing protein
DAB: 3,3′-Diaminobenzidine tetrahydrochloride hydrate
GA: Gibberellic acid
GFP: Green fluorescent protein
GST: Glutathione S-transferase
LUC: Luciferase
MBP: Maltose-binding protein
mCherry: mCherry fluorescent protein
MeJA: methyl jasmonate
MV: Methyl viologen dichloride
NBT: Nitroblue tetrazolium
NOX: NADPH oxidase
PEG: Polyethylene glycol
RBOH: Respiratory burst oxidase homolog
RLK: Receptor-like protein kinase
RLCK: Receptor-like cytoplasmic kinase
ROS: Reactive oxygen species
RPK: Receptor protein kinases
SA: Salicylic acid
WT: Wild-type
