# Correction to: Rice Carbohydrate‑Binding Malectin‑Like Protein, OsCBM1, Contributes to Drought‑Stress Tolerance by Participating in NADPH Oxidase‑Mediated ROS Production

**DOI:** 10.1186/s12284-022-00551-x

**Published:** 2022-01-11

**Authors:** Xiu‑Qing Jing, Wen‑Qiang Li, Meng‑Ru Zhou, Peng‑Tao Shi, Ran Zhang, Abdullah Shalmani, Izhar Muhammad, Gang‑Feng Wang, Wen‑Ting Liu, Kun‑Ming Chen

**Affiliations:** 1 State Key Laboratory of Crop Stress Biology in Arid Area, College of Life Sciences, Northwest A&F University, Yangling, 712100 Shaanxi China; 2 Department of Biology, Taiyuan Normal University, Taiyuan, 030619 Shanxi China

## Correction to: Rice (2021) 14:100 10.1186/s12284-021-00541-5

Unfortunately in the original version of the article, the Figure 5D was published incorrectly. The corrected figure 5 is given below.

**Fig. 5:**
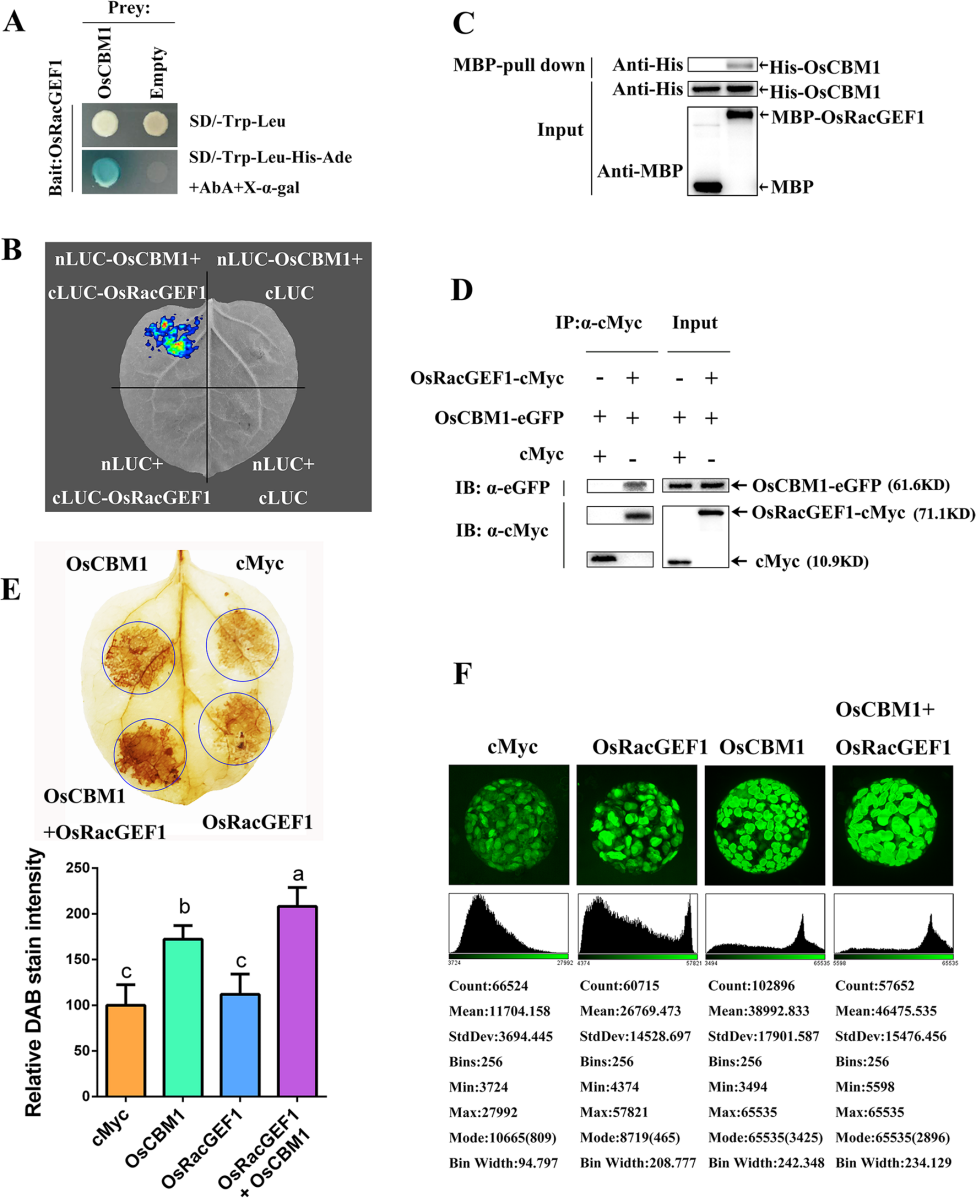
OsCBM1 interacts with OsRacGEF1 and their coexpression enhanced reactive oxygen species (ROS) production. **A** Split-ubiquitin yeast two-hybrid assays of the “bait” pGBKT7-OsRacGEF1 with the “prey” pGADT7-OsCBM1. **B** Firefly luciferase complementation imaging (LCI) assay. **C** MBP-pull down assay, showing the interaction of OsCBM1 with OsRacGEF1 in vitro. **D** Co-immunoprecipitation (Co-IP) assay, showing the physical interaction of OsCBM1-eGFP with OsRacGEF1-6 × cMyc in vivo. **E** Transient coexpression of OsCBM1 and OsRacGEF1 in the leaves of *Nicotiana benthamiana*. The 3,3′-diaminobenzidine (DAB)-stained *N. benthamiana* leaves were transiently transformed with cMyc (P35S-cMyc), OsCBM1 (P35S-OsCBM1), OsRacGEF1 (P35S-OsRacGEF1), and their combination, respectively. The DAB staining intensity in situ ROS levels of agroinfiltrated *N. benthamiana* leaves in each treatment was calculated based on the stain intensity of the control cMyc. Bars annotated with different letters represent values that are significantly different (*p* ≤ 0.05) according to a one-way ANOVA. **F** Detection of ROS production by H2DCFDA fluorescent probe in *N. benthamiana* protoplasts isolated from the leaves of *N. benthamiana* agroinfiltrated by different vectors. Bars = 10 μm. The intensity of fluorescent signals was calculated with ImageJ 1.8.0 software and presented with scatter diagrams (the bottom images in **F**)

The original article has been corrected.

