# Supplementary figures and images for: Rice Carbohydrate-Binding Malectin-Like Protein, OsCBM1, Contributes to Drought-Stress Tolerance by Participating in NADPH Oxidase-Mediated ROS Production
Source: Rice (N Y). 2021 Dec 7;14:100. doi: 10.1186/s12284-021-00541-5 (PMC8651890; doi:10.1186/s12284-021-00541-5)

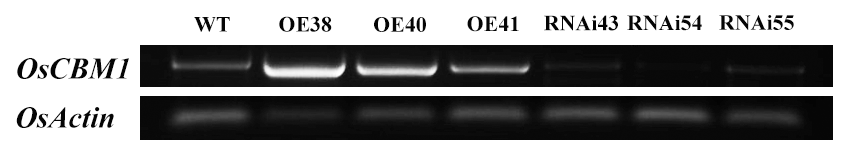

Supplement: Supplementary file 1 — Additional file 1: Figure S1. Expression levels of OsCBM1 in the wild-type (WT), OsCBM1-overexpressing (OE), and RNA interference (RNAi) plants at tillering stages detected by semi-quantitative RT-PCR, using OsActin1 as the internal control. [file 12284_2021_541_MOESM1_ESM.tif]

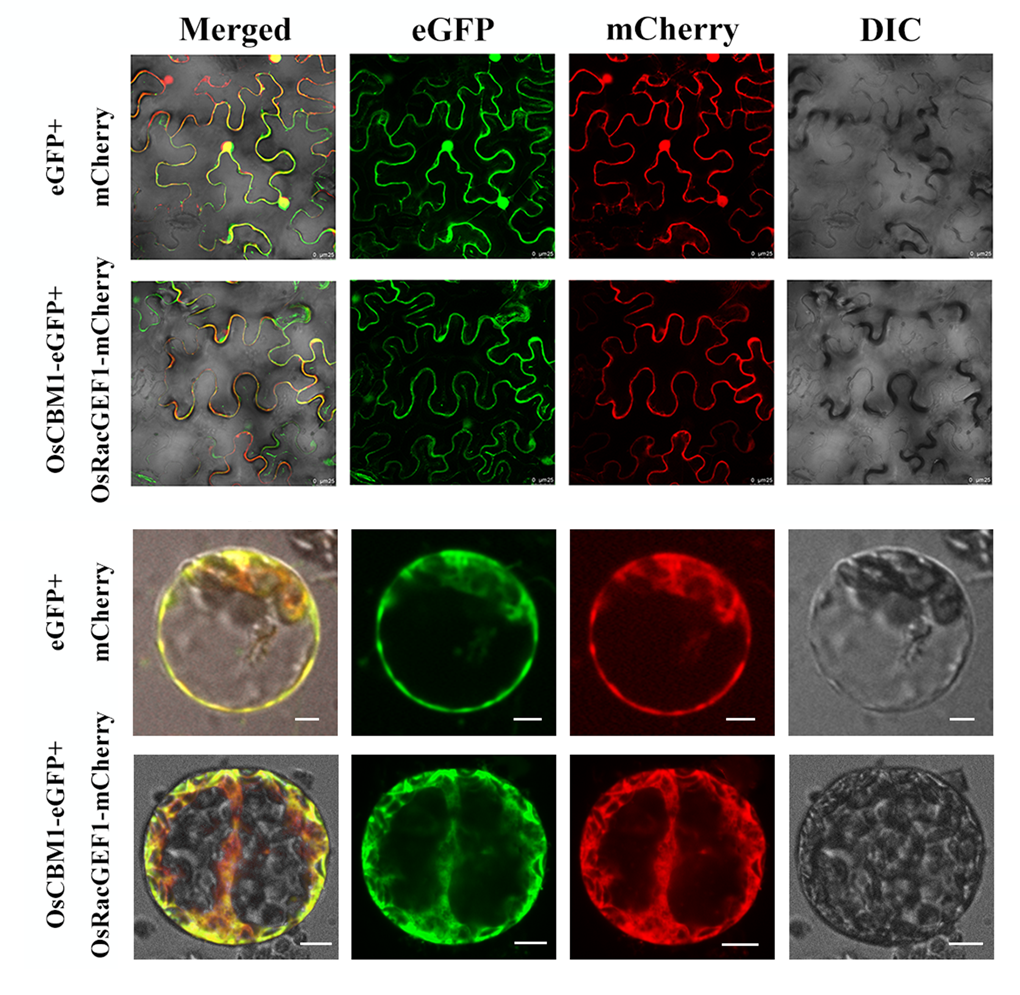

Supplement: Supplementary file 3 — Additional file 3: Figure S3. Co-localization analysis of OsCBM1 and OsRacGEF1. Subcellular localization of OsCBM1 and OsRacGEF1 were detected using the Agrobacterium-mediated transformation with both N. benthamiana epidermal cells and protoplasts, respectively. The plasmids containing eGFP, mCherry, OsCBM1-eGFP, or OsRacGEF1-mCherry genes were under the control of the CaMV 35S promoter. Bars = 10 μm. DIC, bright field. [file 12284_2021_541_MOESM3_ESM.tif]

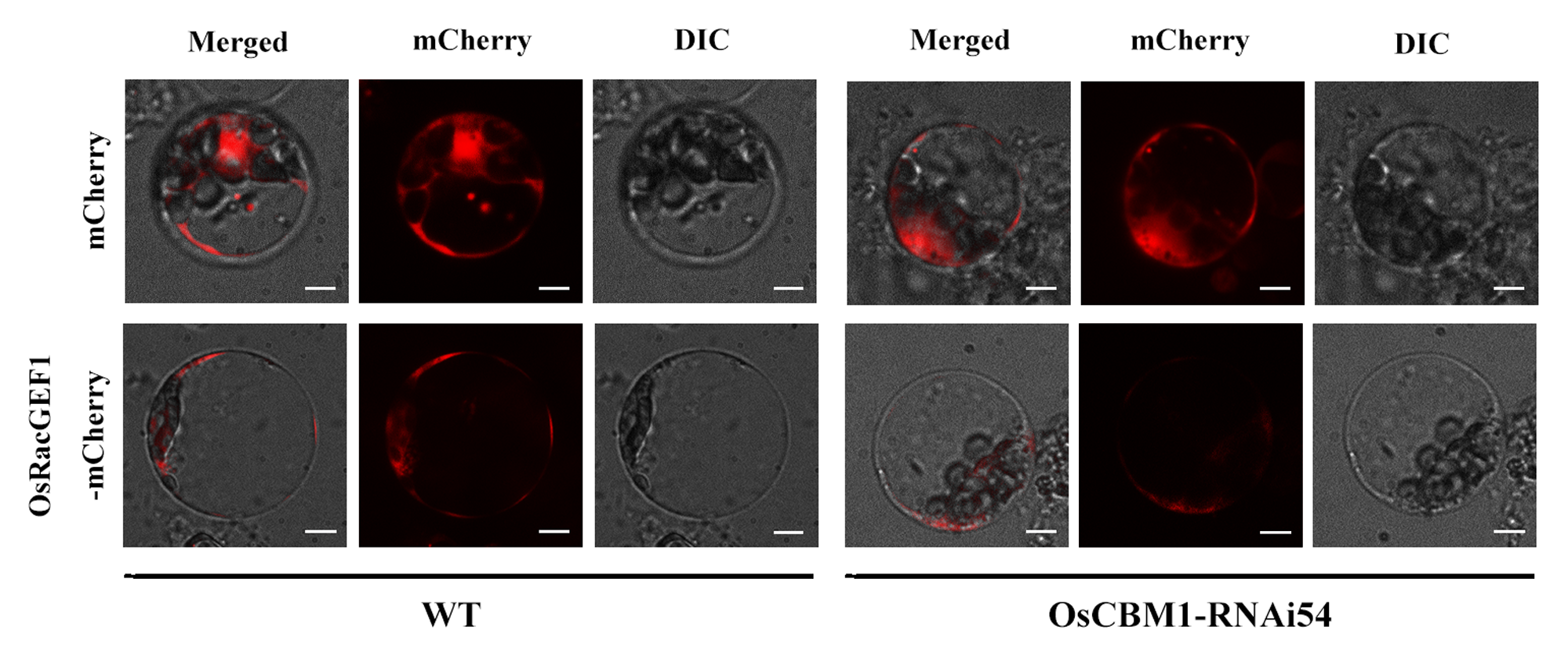

Supplement: Supplementary file 4 — Additional file 4: Figure S4. Subcellular localization of OsRacGEF1 in protoplasts of the OsCBM1-RNAi54 transgenic plants and wild type (WT). Subcellular localization of OsRacGEF1 was analyzed with a rice protoplast transient transformation system (Bars = 10 μm). The plasmid containing mCherry and OsRacGEF1-mCherry genes were under the control of the CaMV 35S promoter. DIC, bright field. [file 12284_2021_541_MOESM4_ESM.tif]

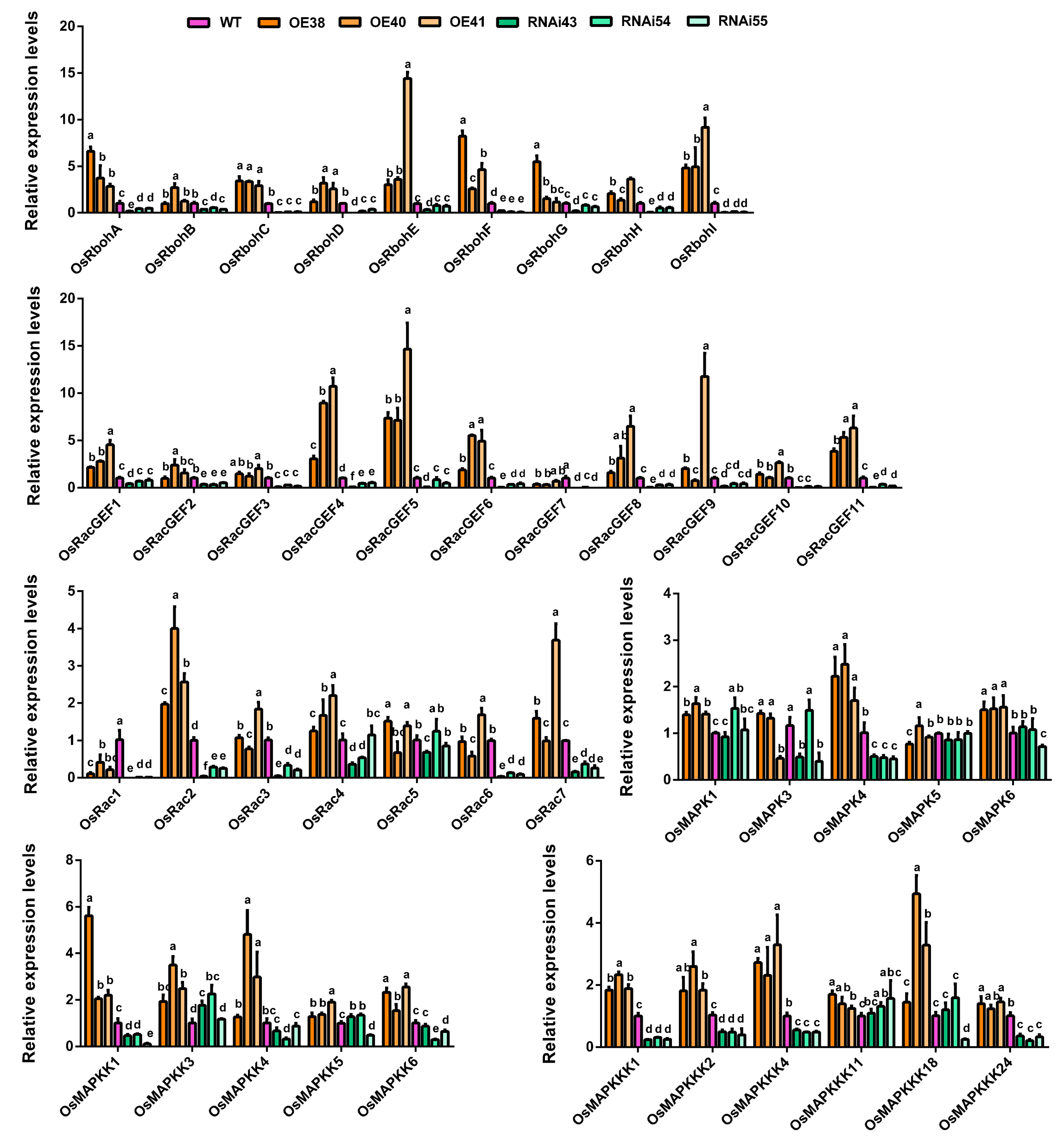

Supplement: Supplementary file 7 — Additional file 7: Figure S5. Transcriptional expression profiles of several protein family genes. Expression levels were detected by qRT-PCR with OsActin1 used as the internal control. Error bars indicate SD from three biological replicates and the bars annotated with different letters represent values that are significantly different (p ≤ 0.05) according to a one-way ANOVA analysis. [file 12284_2021_541_MOESM7_ESM.tif]
