# Supplementary material for: Rice Carbohydrate-Binding Malectin-Like Protein, OsCBM1, Contributes to Drought-Stress Tolerance by Participating in NADPH Oxidase-Mediated ROS Production
Source: Rice (N Y). 2021 Dec 7;14:100. doi: 10.1186/s12284-021-00541-5 (PMC8651890; doi:10.1186/s12284-021-00541-5)
Supplement: Supplementary file 6 — Additional file 6: Table S2. Several gene groups that are differentially expressed in the OsCBM1-downexpressing RNAi54 plants as compared to WT. [file 12284_2021_541_MOESM6_ESM.docx]

**Additional file 6:Table S2 Several gene groups that differentially expressed in the OsCBM1-downexpressing RNAi54 plants as compared to WT.**

| **RAP_Locus** | **MSU_Locus** | **Description** | **Fold change** | **p-value** | **Regulation** |
| --- | --- | --- | --- | --- | --- |
| **Gene encoding ATPase/GTPase** | | | | | |
| Os12g0471100 | LOC_Os12g28590 | ATPase 2, putative, expressed | 14.78 | 6.73E-05 | Down |
| Os12g0639200 | LOC_Os12g44190 | ATPase 3, putative, expressed | 11.96 | 2.97E-06 | Down |
| Os12g0431100 | LOC_Os12g24320 | ATPase 3, putative, expressed | 6.96 | 3.10E-05 | Down |
| Os12g0639500 | LOC_Os12g44220 | ATPase 2, putative, expressed | 5.86 | 1.03E-02 | Down |
| Os02g0537400 | LOC_Os02g33420 | ATPase, AAA family protein, expressed | 5.00 | 6.89E-05 | Down |
| Os06g0565900 | LOC_Os06g36990 | Phospholipid-transporting ATPase 4, putative, expressed | 3.46 | 3.69E-16 | Down |
| Os03g0802500 | LOC_Os03g58790 | ATPase, putative, expressed | 3.23 | 9.05E-04 | Down |
| Os03g0203700 | LOC_Os03g10640 | Calcium-transporting ATPase, plasma membrane-type, putative, expressed | 2.75 | 1.29E-04 | Down |
| Os03g0326000 | LOC_Os03g20949 | Phospholipid-transporting ATPase, putative, expressed | 2.21 | 5.00E-03 | Down |
| Os05g0120700 | LOC_Os05g02940 | Calcium-transporting ATPase 2, endoplasmic reticulum-type, putative, expressed | 2.12 | 6.91E-06 | Down |
| Os11g0485200 | LOC_Os11g29490 | Plasma membrane ATPase, putative, expressed | 2.01 | 4.83E-04 | Up |
| Os09g0521500 | LOC_Os09g34970 | Arsenical pump-driving ATPase, putative, expressed | 2.08 | 3.37E-05 | Up |
| Os11g0446500 | LOC_Os11g25980 | Phospholipid-transporting ATPase 2, putative, expressed | 2.12 | 3.26E-04 | Up |
| Os04g0353000 | LOC_Os04g28460 | Phospholipid-transporting ATPase 2, putative, expressed | 2.18 | 4.94E-08 | Up |
| Os02g0697600 | LOC_Os02g46990 | AAA-type ATPase family protein, putative, expressed | 2.26 | 3.87E-05 | Up |
| Os09g0120800 | LOC_Os09g03310 | Phospholipid-transporting ATPase 2, putative, expressed | 2.33 | 2.83E-09 | Up |
| Os07g0191200 | LOC_Os07g09340 | Plasma membrane ATPase, putative, expressed | 2.40 | 6.10E-03 | Up |
| Os02g0745000 | LOC_Os02g51100 | Arsenical pump-driving ATPase, putative, expressed | 2.42 | 7.85E-05 | Up |
| Os02g0749150 | LOC_Os02g51400 | AAA family ATPase, putative, expressed | 2.46 | 6.06E-07 | Up |
| Os06g0109400 | LOC_Os06g01980 | AAA family ATPase, putative, expressed | 2.64 | 8.24E-11 | Up |
| Os02g0706500 | LOC_Os02g47760 | AAA-type ATPase family protein, putative, expressed | 2.79 | 1.57E-04 | Up |
| Os12g0292900 | LOC_Os12g19530 | ATP-binding region, ATPase-like domain-containing protein, putative, expressed | 3.00 | 1.04E-08 | Up |
| Os04g0658300 | LOC_Os04g56320 | AAA-type ATPase family protein, putative, expressed | 3.45 | 3.60E-08 | Up |
| Os08g0379200 | LOC_Os08g29150 | Phospholipid-transporting ATPase, putative, expressed | 3.83 | 5.33E-06 | Up |
| Os06g0225900 | LOC_Os06g12160 | AAA-type ATPase family protein, putative, expressed | 3.85 | 8.36E-10 | Up |
| Os03g0264800 | LOC_Os03g15810 | AAA-type ATPase family protein, putative, expressed | 4.08 | 9.70E-04 | Up |
| Os07g0192000 | LOC_Os07g09420 | ATPase, putative, expressed | 5.29 | 7.82E-03 | Up |
| Os03g0281900 | LOC_Os03g17350 | ATP binding cassette transporter | 4.53 | 6.30E-03 | Down |
| Os07g0500300 | LOC_Os07g31720 | GTPase activating protein, putative, expressed | 3.81 | 1.30E-07 | Down |
| Os03g0854100 | LOC_Os03g63710 | GTPase-activating protein, putative, expressed | 2.81 | 3.16E-10 | Down |
| Os03g0323500 | LOC_Os03g20720 | GTPase-activating protein, putative, expressed | 2.24 | 2.58E-06 | Down |
| Os11g0592700 | LOC_Os11g38020 | GTPase of unknown function domain containing protein, putative, expressed | 2.20 | 1.05E-07 | Up |
| Os03g0816400 | LOC_Os03g60180 | GTPase of unknown function domain containing protein, putative, expressed | 2.53 | 7.26E-03 | Up |
| Os05g0430400 | LOC_Os05g35540 | GTPase of unknown function domain containing protein, putative, expressed | 2.63 | 4.19E-13 | Up |
| Os03g0647500 | LOC_Os03g44530 | GTPase of unknown function domain containing protein, putative, expressed | 2.65 | 5.88E-04 | Up |
| Os08g0207600 | LOC_Os08g10649 | GTPase of unknown function domain containing protein, putative, expressed | 3.13 | 1.30E-04 | Up |
| Os08g0407000 | LOC_Os08g31460 | GTPase of unknown function domain containing protein, putative, expressed | 3.30 | 2.75E-07 | Up |
| Os01g0375000 | LOC_Os01g27730 | GTPase of unknown function domain containing protein, putative, expressed | 6.51 | 1.90E-17 | Up |
| Os04g0561200 | LOC_Os04g47330 | Rho-GTPase-activating protein-related, putative, expressed | 11.43 | 2.53E-04 | Up |
| Os08g0199300 | LOC_Os08g09940 | GTPase | 2.15 | 7.04E-11 | Up |
| **Gene encoding Rapid ALkalinization Factor RALF family protein** | | | | | |
| Os01g0357900 | LOC_Os01g25540 | RALFL6 - Rapid ALkalinization Factor RALF family protein precursor, expressed | 2.37 | 7.82E-05 | Down |
| Os10g0569400 | LOC_Os10g41980 | RALFL26 - Rapid ALkalinization Factor RALF family protein precursor, expressed | 4.18 | 7.68E-03 | Up |
| Os10g0569500 | LOC_Os10g41999 | RALFL27 - Rapid ALkalinization Factor RALF family protein precursor, expressed | 6.98 | 8.28E-05 | Up |
| Os12g0218300 | LOC_Os12g11660 | RALFL45 - Rapid ALkalinization Factor RALF family protein precursor, expressed | 112.98 | 1.98E-16 | Down |
| Os12g0221600 | LOC_Os12g12000 | RALFL46 - Rapid ALkalinization Factor RALF family protein precursor, expressed | 96.69 | 3.02E-15 | Down |
| Os12g0571600 | LOC_Os12g38350 | RALFL43 - Rapid ALkalinization Factor RALF family protein precursor, expressed | 30.64 | 1.92E-07 | Down |
| Os12g0571700 | LOC_Os12g38360 | RALFL31 - Rapid ALkalinization Factor RALF family protein precursor, expressed | 22.06 | 1.20E-06 | Down |
| Os04g0384000 | LOC_Os04g31484 | RALFL20 - Rapid ALkalinization Factor RALF family protein precursor, expressed | 22.03 | 5.02E-06 | Down |
| Os04g0357400 | LOC_Os04g28830 | RALFL34 - Rapid ALkalinization Factor RALF family protein precursor, putative, expressed | 19.19 | 1.43E-05 | Down |
| Os04g0383700 | LOC_Os04g31460 | RALFL39 - Rapid ALkalinization Factor RALF family protein precursor, expressed | 11.76 | 7.51E-05 | Down |
| Os04g0383100 | LOC_Os04g31430 | RALFL30 - Rapid ALkalinization Factor RALF family protein precursor, putative, expressed | 7.66 | 5.18E-03 | Down |
| Os01g0358100 | LOC_Os01g25560 | RALFL7 - Rapid ALkalinization Factor RALF family protein precursor, expressed | 7.33 | 6.84E-05 | Down |
| Os11g0448700 | LOC_Os11g26190 | RALFL23 - Rapid ALkalinization Factor RALF family protein precursor, expressed | 6.30 | 1.24E-02 | Down |
| **Gene encoding lectin-like receptor kinase family protein** | | | | | |
| Os04g0202800 | LOC_Os04g12600 | Lectin-like receptor kinase | 59.92 | 1.73E-11 | Down |
| Os04g0330200 | LOC_Os04g26320 | Mannose-binding lectin, putative, expressed | 232.73 | 6.70E-24 | Down |
| Os08g0124100 | LOC_Os08g03002 | Lectin-like receptor kinase 1, putative, expressed | 27.87 | 2.05E-07 | Down |
| Os07g0131500 | LOC_Os07g03950 | Lectin-like receptor kinase, putative, expressed | 18.01 | 1.37E-05 | Down |
| Os08g0124500 | LOC_Os08g03020 | Lectin-like receptor kinase 1, putative, expressed | 14.79 | 1.06E-06 | Down |
| Os08g0124700 | LOC_Os08g03070 | Lectin-like receptor kinase 1, putative, expressed | 13.59 | 2.06E-05 | Down |
| Os04g0123800 | LOC_Os04g03380 | Legume lectins beta domain containing protein, putative, expressed | 12.67 | 2.26E-04 | Down |
| Os05g0143600 | LOC_Os05g05170 | Jacalin-like lectin domain containing protein, expressed | 6.83 | 8.13E-03 | Down |
| Os02g0712700 | LOC_Os02g48210 | Lectin-like protein kinase, putative, expressed | 6.75 | 3.02E-07 | Down |
| Os07g0262600 | LOC_Os07g15930 | Legume lectins beta domain containing protein, expressed | 5.93 | 1.47E-02 | Down |
| Os05g0125200 | LOC_Os05g03450 | Lectin protein kinase family protein, putative, expressed | 5.38 | 5.99E-05 | Down |
| Os04g0109500 | LOC_Os04g01910 | Jegume lectins beta domain containing protein, putative, expressed | 4.77 | 6.93E-03 | Down |
| Os06g0579800 | LOC_Os06g38160 | Jegume lectins beta domain containing protein, putative, expressed | 3.59 | 4.46E-03 | Down |
| Os10g0132300 | LOC_Os10g04270 | Jacalin-like lectin domain containing protein, expressed | 2.88 | 1.42E-07 | Down |
| Os03g0399800 | LOC_Os03g28160 | Jacalin-like lectin domain containing protein, expressed | 2.86 | 6.80E-09 | Down |
| Os10g0442000 | LOC_Os10g30540 | Lectin-like receptor kinase, putative, expressed | 2.14 | 1.78E-02 | Down |
| Os03g0823000 | LOC_Os03g60810 | Lectin-like receptor kinase, putative, expressed | 2.13 | 2.40E-06 | Down |
| Os09g0454900 | LOC_Os09g28180 | D-mannose binding lectin family protein, expressed | 2.10 | 9.38E-03 | Down |
| Os05g0508400 | LOC_Os05g43240 | Jacalin-like lectin domain containing protein, expressed | 2.19 | 4.75E-03 | Up |
| Os01g0783900 | LOC_Os01g57490 | D-mannose binding lectin family protein, expressed | 2.38 | 2.11E-04 | Up |
| Os07g0283050 | LOC_Os07g18230 | Lectin-like receptor kinase, putative, expressed | 3.51 | 1.28E-06 | Up |
| Os04g0303100 | LOC_Os04g23700 | Lectin protein kinase family protein, putative, expressed | 3.74 | 8.81E-04 | Up |
| Os06g0551800 | LOC_Os06g35850 | Lectin protein kinase family protein, putative, expressed | 56.17 | 6.23E-44 | Up |
| **Gene encoding ankyrin repeat-containing protein** | | | | | |
| Os11g0141900 | LOC_Os11g04600 | BTBA5 - Bric-a-Brac,Tramtrack, Broad Complex BTB domain with Ankyrin repeat region, expressed | 10.43 | 1.03E-06 | Down |
| Os07g0512100 | LOC_Os07g32790 | Ankyrin repeat domain containing protein, expressed | 9.08 | 8.29E-06 | Down |
| Os03g0275300 | LOC_Os03g16780 | Ankyrin repeat family protein, putative, expressed | 6.84 | 7.07E-03 | Down |
| Os01g0829100 | LOC_Os01g61330 | Ankyrin homolog precursor, putative, expressed | 6.66 | 3.32E-15 | Down |
| Os03g0655500 | LOC_Os03g45290 | Ankyrin repeat domain-containing protein 50, putative, expressed | 6.55 | 1.02E-02 | Down |
| Os12g0138500 | LOC_Os12g04410 | BTBA6 - Bric-a-Brac,Tramtrack, Broad Complex BTB domain with Ankyrin repeat region, expressed | 3.63 | 5.02E-04 | Down |
| Os09g0337300 | LOC_Os09g16760 | Ankyrin repeat family protein, putative, expressed | 2.89 | 8.65E-03 | Down |
| Os08g0542700 | LOC_Os08g42960 | Ankyrin repeat-containing protein, putative, expressed | 2.21 | 1.57E-04 | Down |
| Os08g0233400 | LOC_Os08g13640 | Ankyrin, putative, expressed | 2.11 | 1.49E-05 | Down |
| Os09g0518500 | LOC_Os09g34280 | Ankyrin repeat-containing protein, putative, expressed | 2.06 | 9.40E-04 | Up |
| Os06g0237000 | LOC_Os06g13000 | Ankyrin repeat domain-containing protein, chloroplast precursor, putative, expressed | 2.42 | 6.59E-05 | Up |
| Os07g0406600 | LOC_Os07g22390 | Ankyrin repeat domain containing protein, expressed | 2.44 | 2.58E-10 | Up |
| Os03g0761200 | LOC_Os03g55330 | Ankyrin repeat domain containing protein, putative, expressed | 3.39 | 2.86E-05 | Up |
| Os01g0189100 | LOC_Os01g09384 | Ankyrin repeat domain containing protein, putative, expressed | 3.92 | 3.48E-03 | Up |
| Os03g0135600 | LOC_Os03g04300 | Ankyrin repeat domain containing protein, expressed | 4.13 | 5.72E-17 | Up |
| **Gene encoding wall-associated receptor kinase** | | | | | |
| Os01g0155800 | LOC_Os01g06310 | Glycine-rich cell wall structural protein precursor, putative, expressed | 195.92 | 1.04E-32 | Down |
| Os04g0366800 | LOC_Os04g29770 | Wall-associated receptor kinase-like 3 precursor, putative, expressed | 76.18 | 2.03E-13 | Down |
| Os11g0691400 | LOC_Os11g46900 | Wall-associated receptor kinase 3 precursor, putative, expressed | 65.83 | 3.02E-14 | Down |
| Os11g0691600 | LOC_Os11g46950 | Wall-associated receptor kinase-like 18 precursor, putative, expressed | 32.63 | 3.01E-08 | Down |
| Os04g0367900 | LOC_Os04g29950 | Wall-associated receptor kinase, putative, expressed | 7.93 | 3.93E-03 | Down |
| Os02g0581900 | LOC_Os02g37109 | Vegetative cell wall protein gp1 precursor, putative, expressed | 5.86 | 4.55E-07 | Down |
| Os04g0371000 | LOC_Os04g30250 | Wall-associated receptor kinase-like 5 precursor, putative, expressed | 4.71 | 1.60E-03 | Down |
| Os08g0378000 | LOC_Os08g29020 | Wall-associated kinase-like 2, putative, expressed | 3.65 | 1.50E-05 | Down |
| Os06g0316300 | LOC_Os06g21140 | Glycine-rich cell wall structural protein precursor, putative, expressed | 2.05 | 4.12E-03 | Up |
| Os03g0378200 | LOC_Os03g26090 | GPI-anchored wall transfer protein 1, putative, expressed | 2.53 | 1.44E-05 | Up |
| Os11g0691100 | LOC_Os11g46860 | Wall-associated receptor kinase-like 4 precursor, putative, expressed | 8.59 | 3.33E-03 | Up |
| Os02g0111600 | LOC_Os02g02120 | Wall-associated kinase gene 11 | 2.80 | 1.50E-04 | Down |
| **Gene encoding PPR repeat domain containing protein** | | | | | |
| Os07g0621100 | LOC_Os07g42880 | PPR repeat containing protein, expressed | 5.25 | 6.92E-03 | Down |
| Os11g0433600 | LOC_Os11g24570 | PPR repeat domain containing protein, putative, expressed | 2.04 | 7.86E-03 | Up |
| Os02g0127600 | LOC_Os02g03530 | PPR repeat domain containing protein, putative, expressed | 2.05 | 2.54E-03 | Up |
| Os05g0294600 | LOC_Os05g22870 | PPR repeat domain containing protein, putative, expressed | 2.13 | 9.54E-05 | Up |
| Os12g0562300 | LOC_Os12g37550 | PPR repeat domain containing protein, putative, expressed | 2.20 | 5.31E-05 | Up |
| Os10g0479200 | LOC_Os10g33874 | PPR repeat domain containing protein, putative, expressed | 2.21 | 1.72E-10 | Up |
| Os10g0422566 | LOC_Os10g28665 | PPR repeat domain containing protein, putative, expressed | 2.21 | 1.47E-02 | Up |
| Os03g0795400 | LOC_Os03g58120 | PPR repeat domain containing protein, putative, expressed | 2.28 | 2.34E-04 | Up |
| Os08g0402600 | LOC_Os08g31110 | PPR repeat domain containing protein, putative, expressed | 2.28 | 1.01E-02 | Up |
| Os02g0273800 | LOC_Os02g17360 | PPR repeat domain containing protein, putative, expressed | 2.30 | 2.34E-05 | Up |
| Os12g0456100 | LOC_Os12g27060 | PPR repeat domain containing protein, putative, expressed | 2.40 | 8.31E-03 | Up |
| Os03g0363700 | LOC_Os03g24880 | PPR repeat containing protein, expressed | 2.44 | 1.87E-03 | Up |
| Os06g0172000 | LOC_Os06g07550 | PPR repeat domain containing protein, putative, expressed | 2.48 | 1.46E-02 | Up |
| Os05g0275100 | LOC_Os05g19390 | PPR repeat containing protein, expressed | 2.52 | 1.50E-03 | Up |
| Os03g0211600 | LOC_Os03g11310 | PPR repeat domain containing protein, putative, expressed | 2.54 | 4.52E-09 | Up |
| Os02g0266200 | LOC_Os02g16650 | PPR repeat domain containing protein, putative, expressed | 2.54 | 2.39E-05 | Up |
| Os04g0618050 | LOC_Os04g52725 | PPR repeat domain containing protein, putative, expressed | 2.56 | 4.41E-03 | Up |
| Os04g0475800 | LOC_Os04g40010 | PPR repeat domain containing protein, putative, expressed | 2.56 | 5.30E-04 | Up |
| Os02g0119700 | LOC_Os02g02740 | PPR repeat containing protein, expressed | 2.56 | 8.93E-03 | Up |
| Os03g0162900 | LOC_Os03g06710 | PPR repeat domain containing protein, putative, expressed | 2.58 | 1.26E-03 | Up |
| Os07g0222700 | LOC_Os07g12140 | PPR repeat domain containing protein, putative, expressed | 2.67 | 6.44E-07 | Up |
| Os03g0309800 | LOC_Os03g19650 | PPR2, putative, expressed | 2.73 | 1.81E-20 | Up |
| Os01g0970900 | LOC_Os01g73950 | PPR repeat containing protein, expressed | 2.77 | 1.37E-03 | Up |
| Os07g0598500 | LOC_Os07g40750 | PPR repeat domain containing protein, putative, expressed | 2.79 | 1.03E-03 | Up |
| Os02g0132600 | LOC_Os02g03970 | PPR repeat domain containing protein, putative, expressed | 2.80 | 2.55E-17 | Up |
| Os07g0549200 | LOC_Os07g36450 | PPR repeat domain containing protein, putative, expressed | 2.87 | 2.40E-03 | Up |
| Os03g0824100 | LOC_Os03g60910 | PPR repeat domain containing protein, putative, expressed | 2.89 | 1.04E-05 | Up |
| Os03g0168400 | LOC_Os03g07220 | PPR repeat domain containing protein, putative, expressed | 2.91 | 1.33E-09 | Up |
| Os10g0116000 | LOC_Os10g02650 | PPR repeat containing protein, expressed | 2.97 | 7.50E-11 | Up |
| Os07g0688100 | LOC_Os07g48850 | PPR repeat containing protein, expressed | 3.03 | 6.29E-04 | Up |
| Os03g0115300 | LOC_Os03g02430 | PPR repeat domain containing protein, putative, expressed | 3.05 | 4.91E-04 | Up |
| Os02g0470000 | LOC_Os02g27070 | PPR repeat domain containing protein, putative, expressed | 3.12 | 1.31E-07 | Up |
| Os09g0473800 | LOC_Os09g29790 | PPR repeat domain containing protein, putative, expressed | 3.13 | 1.69E-03 | Up |
| Os09g0473800 | LOC_Os09g29790 | PPR repeat domain containing protein, putative, expressed | 3.13 | 1.78E-07 | Up |
| Os05g0315100 | LOC_Os05g25060 | PPR repeat domain containing protein, putative, expressed | 3.21 | 2.46E-07 | Up |
| Os02g0750400 | LOC_Os02g51480 | PPR repeat domain containing protein, putative, expressed | 3.32 | 4.05E-19 | Up |
| Os07g0495300 | LOC_Os07g31310 | PPR repeat domain containing protein, putative, expressed | 3.37 | 3.00E-07 | Up |
| Os03g0284900 | LOC_Os03g17634 | PPR repeat domain containing protein, putative, expressed | 3.38 | 2.02E-11 | Up |
| Os05g0481800 | LOC_Os05g40320 | PPR repeat domain containing protein, putative, expressed | 3.43 | 1.93E-08 | Up |
| Os05g0365500 | LOC_Os05g30240 | PPR repeat domain containing protein, putative, expressed | 3.43 | 1.98E-05 | Up |
| Os04g0488500 | LOC_Os04g41140 | PPR repeat containing protein, expressed | 3.47 | 2.45E-05 | Up |
| Os06g0111300 | LOC_Os06g02120 | PPR repeat domain containing protein, putative, expressed | 3.56 | 1.62E-14 | Up |
| Os01g0757700 | LOC_Os01g55290 | PPR repeat domain containing protein, putative, expressed | 3.58 | 2.75E-10 | Up |
| Os03g0216300 | LOC_Os03g11690 | PPR repeat containing protein, expressed | 3.66 | 7.62E-03 | Up |
| Os08g0369200 | LOC_Os08g28180 | PPR repeat domain containing protein, putative, expressed | 3.90 | 4.40E-08 | Up |
| Os02g0611400 | LOC_Os02g39820 | PPR repeat domain containing protein, putative, expressed | 4.03 | 1.59E-08 | Up |
| Os07g0548300 | LOC_Os07g36390 | PPR repeat containing protein, expressed | 4.20 | 2.19E-33 | Up |
| Os07g0513200 | LOC_Os07g32900 | PPR repeat containing protein, expressed | 4.23 | 1.75E-04 | Up |
| Os02g0644600 | LOC_Os02g43080 | PPR repeat domain containing protein, putative, expressed | 4.52 | 2.26E-09 | Up |
| Os12g0170100 | LOC_Os12g07260 | PPR repeat domain containing protein, putative, expressed | 4.56 | 3.51E-13 | Up |
| Os06g0690900 | LOC_Os06g47570 | PPR repeat containing protein, expressed | 5.18 | 1.81E-08 | Up |
| Os05g0145600 | LOC_Os05g05320 | PPR repeat domain containing protein, putative, expressed | 5.22 | 9.03E-17 | Up |
| Os01g0280400 | LOC_Os01g17320 | PPR repeat domain containing protein, putative, expressed | 5.94 | 6.28E-04 | Up |
| Os07g0615000 | LOC_Os07g42354 | PPR repeat domain containing protein, putative, expressed | 6.39 | 1.51E-14 | Up |
| Os04g0544400 | LOC_Os04g46010 | PPR repeat domain containing protein, putative, expressed | 7.54 | 1.34E-26 | Up |
| Os01g0263400 | LOC_Os01g15840 | PPR repeat containing protein, expressed | 14.27 | 1.39E-09 | Up |
| **Gene encoding stress-activated protein kinase** | | | | | |
| Os01g0686800 | LOC_Os01g49290 | Receptor for Activated C-kinase 1, RACK1 | 5.22 | 3.29E-15 | Up |
| Os05g0552300 | LOC_Os05g47890 | Receptor for activated C kinase 1B, RACK1B | 4.34 | 4.48E-18 | Up |
| Os01g0686800 | LOC_Os01g49290 | Receptor for Activated C-kinase 1, RACK1 | 5.22 | 3.29E-15 | Up |
| Os04g0432000 | LOC_Os04g35240 | stress/ABA-activated protein kinase 7, OsSAPK7 | 4.40 | 8.43E-10 | Down |
| Os10g0564500 | LOC_Os10g41490 | Stress-Activated Protein Kinase 3, OsSAPK3 | 3.41 | 4.46E-12 | Down |
| Os01g0869900 | LOC_Os01g64970 | Stress-Activated Protein Kinase 4, OsSAPK4 | 3.11 | 4.87E-08 | Down |
| **Gene encoding proteins related to the morphology of panicle** | | | | | |
| Os01g0197700 | LOC_Os01g10110 | Grain number 1a, cytokinin oxidase 2, Gn1a/OsCKX2 | 3.56 | 5.15E-03 | Up |
| Os05g0482400 | LOC_Os05g40384 | Elongated uppermost internode-1, EUI1 | 2.64 | 4.99E-04 | Down |
| Os11g0247300 | LOC_Os11g14220 | Alpha-tubulin protein, Cell elongation and division, OS-TubA2 | 3.95 | 1.24E-10 | Down |
| Os07g0616000 | LOC_Os07g42410 | Regulation of panicle erectness, panicle length and grain size | 2.40 | 1.98E-06 | Down |
| Os01g0718300 | LOC_Os01g52050 | Similar to Brassinosteroid-insensitive 1, Os BRI1 | 2.69 | 1.58E-04 | Down |
| Os06g0649600 | LOC_Os06g44034 | Multi-tiller and semi-dwarf | 2.60 | 3.67E-05 | Down |
| Os01g0197700 | LOC_Os01g10110 | Cytokinin oxidase/dehydrogenase 2 | 3.56 | 5.15E-03 | Up |
| **Gene encoding stress associated protein gene** | | | | | |
| Os02g0669100 | LOC_Os02g44870 | Dehydration-stress inducible protein 1 | 2.14 | 1.15E-04 | Down |
| Os07g0569700 | LOC_Os07g38240 | C2H2 transcription factor, stress associated protein 16, OsSAP16 | 2.05 | 3.50E-04 | Down |
| Os03g0793000 | LOC_Os03g57900 | Stress associated protein 7, OsSAP7 | 2.03 | 8.74E-04 | Down |
| Os01g0233000 | LOC_Os01g13210 | Salt stress root protein RS1, putative, expressed | 2.79 | 5.19E-04 | Down |
| Os03g0179400 | LOC_Os03g08170 | Drought-inducible receptor-like cytoplasmic kinase, OsRLCK103 | 3.25 | 1.70E-07 | Down |
| Os03g0286900 | LOC_Os03g17790 | Drought resistance, rare cold-inducible 2-5, OsRCI2-5 | 2.82 | 5.17E-03 | Down |
| Os01g0263300 | LOC_Os01g15830 | Similar to Peroxidase 72 precursor | 4.69 | 5.12E-05 | Down |
| Os03g0150800 | LOC_Os03g05640 | High affinity phosphate transporter 2, OsPT2 | 4.15 | 2.48E-03 | Down |
| Os09g0325700 | LOC_Os09g15670 | Protein phosphatase 2C, Abiotic stress response, Early panicle development,   PP2C1 | 4.09 | 7.16E-09 | Down |
| Os05g0380900 | LOC_Os05g31620 | Calmodulin-like protein 15, OsCML15 | 3.85 | 2.14E-06 | Down |
| **Gene encoding receptor protein kinase** | | | | | |
| Os09g0355400 | LOC_Os09g18594 | Protein kinase domain containing protein, expressed | 1514.04 | 3.57E-49 | Down |
| Os02g0615800 | LOC_Os02g40240 | Leucine-rich repeat receptor protein kinase | 1075.27 | 3.41E-80 | Down |
| Os11g0695000 | LOC_Os11g47240 | Leucine-rich repeat receptor protein kinase EXS precursor, putative, expressed | 367.37 | 2.03E-33 | Down |
| Os09g0356200 | LOC_Os09g19160 | Serine/threonine-protein kinase, putative, expressed | 239.01 | 3.01E-23 | Down |
| Os11g0640300 | LOC_Os11g42070 | Leucine Rich Repeat family protein, expressed | 147.66 | 5.83E-19 | Down |
| Os11g0688832 | LOC_Os11g46200 | Leucine Rich Repeat family protein, expressed | 76.23 | 2.76E-13 | Down |
| Os01g0117500 | LOC_Os01g02810 | Resistance-related receptor-like kinase, putative, expressed | 40.61 | 2.49E-09 | Down |
| Os03g0228800 | LOC_Os03g12730 | Leucine-rich repeat receptor protein kinase | 31.62 | 1.40E-21 | Down |
| Os11g0625900 | LOC_Os11g40970 | Receptor-like protein kinase precursor, putative, expressed | 31.31 | 6.30E-08 | Down |
| Os05g0550700 | LOC_Os05g47750 | Ser/Thr protein kinase, putative, expressed | 18.48 | 7.32E-07 | Down |
| Os10g0533150 | LOC_Os10g38920 | Protein kinase domain containing protein, expressed | 18.41 | 5.44E-08 | Down |
| Os01g0170300 | LOC_Os01g07560 | Receptor-like protein kinase 2 precursor, putative, expressed | 18.09 | 2.83E-09 | Down |
| Os04g0649700 | LOC_Os04g55620 | Receptor kinase, putative, expressed | 16.80 | 3.90E-12 | Down |
| Os11g0689000 | LOC_Os11g46220 | Senescence-induced receptor-like serine/threonine-protein kinase precursor, putative, expressed | 16.78 | 2.56E-05 | Down |
| Os03g0127700 | LOC_Os03g03570 | Leucine-rich repeat transmembrane protein kinase, putative, expressed | 16.70 | 6.97E-13 | Down |
| Os02g0182600 | LOC_Os02g08530 | Protein kinase family protein, putative, expressed | 13.01 | 4.26E-14 | Down |
| Os05g0478300 | LOC_Os05g40050 | Receptor-like protein kinase 2 precursor, putative, expressed | 12.47 | 1.38E-09 | Down |
| Os10g0492400 | LOC_Os10g35040 | Receptor kinase like protein, putative, expressed | 12.42 | 2.48E-12 | Down |
| Os11g0695800 | LOC_Os11g47310 | Receptor kinase-like protein, identical, putative, expressed | 12.23 | 2.85E-04 | Down |
| Os03g0335500 | LOC_Os03g21730 | Receptor-like protein kinase precursor, putative, expressed | 12.17 | 2.83E-04 | Down |
| Os07g0152200 | LOC_Os07g05740 | Receptor-like protein kinase 2 precursor, putative, expressed | 12.13 | 2.71E-08 | Down |
| Os12g0108100 | LOC_Os12g01740 | Serine/threonine-protein kinase, putative, expressed | 11.86 | 3.40E-15 | Down |
| Os06g0693200 | LOC_Os06g47820 | Protein kinase domain containing protein, expressed | 11.24 | 1.09E-24 | Down |
| Os12g0632800 | LOC_Os12g43640 | Receptor-like protein kinase HAIKU2 precursor, putative, expressed | 11.20 | 2.22E-19 | Down |
| Os05g0406800 | LOC_Os05g33690 | Receptor-like protein kinase precursor, putative, expressed | 9.83 | 1.30E-03 | Down |
| Os01g0162500 | LOC_Os01g06890 | Leucine-rich repeat family protein, putative, expressed | 9.80 | 1.19E-03 | Down |
| Os02g0615500 | LOC_Os02g40200 | Receptor-like protein kinase precursor, putative, expressed | 9.75 | 1.27E-03 | Down |
| Os12g0638100 | LOC_Os12g44090 | Leucine-rich repeat family protein, putative, expressed | 9.72 | 1.40E-05 | Down |
| Os11g0606200 | LOC_Os11g39290 | Leucine Rich Repeat family protein, expressed | 8.83 | 1.74E-04 | Down |
| Os11g0108800 | LOC_Os11g01740 | Serine/threonine-protein kinase, putative, expressed | 8.68 | 7.04E-05 | Down |
| Os08g0124000 | LOC_Os08g02996 | Receptor-like kinase, putative, expressed | 7.87 | 2.42E-08 | Down |
| Os11g0653300 | LOC_Os11g43250 | Leucine Rich Repeat family protein, expressed | 7.84 | 4.35E-04 | Down |
| Os03g0556600 | LOC_Os03g35600 | Serine/threonine-protein kinase receptor precursor, putative, expressed | 7.45 | 2.27E-09 | Down |
| Os03g0720166 | LOC_Os03g51060 | Inactive receptor kinase At2g26730 precursor, putative, expressed | 7.15 | 6.41E-03 | Down |
| Os03g0184400 | LOC_Os03g08610 | Leucine-rich repeat family protein, putative, expressed | 6.91 | 7.46E-03 | Down |
| Os01g0323000 | LOC_Os01g21960 | Serine/threonine-protein kinase, putative, expressed | 6.82 | 2.27E-03 | Down |
| Os09g0442100 | LOC_Os09g27010 | Tyrosine protein kinase domain containing protein, putative, expressed | 6.76 | 1.35E-07 | Down |
| Os08g0148300 | LOC_Os08g05290 | Receptor-like protein kinase 5 precursor, putative, expressed | 6.54 | 3.65E-04 | Down |
| Os05g0550800 | LOC_Os05g47770 | Serine/threonine-protein kinase At1g18390 precursor, putative, expressed | 6.45 | 7.63E-07 | Down |
| Os01g0149700 | LOC_Os01g05640 | Receptor-like protein kinase 5 precursor, putative, expressed | 6.38 | 1.61E-05 | Down |
| Os01g0138400 | LOC_Os01g04580 | Ser/Thr protein kinase, putative, expressed | 6.31 | 3.72E-03 | Down |
| Os01g0117400 | LOC_Os01g02800 | Receptor-like kinase ARK1AS, putative, expressed | 6.25 | 1.16E-04 | Down |
| Os08g0442700 | LOC_Os08g34380 | Leucine-rich repeat receptor protein kinase | 6.04 | 1.83E-09 | Down |
| Os01g0941800 | LOC_Os01g71420 | Ser/Thr protein phosphatase family protein, putative, expressed | 5.92 | 1.71E-03 | Down |
| Os06g0186300 | LOC_Os06g08710 | Receptor-like protein kinase precursor, putative, expressed | 5.90 | 1.17E-03 | Down |
| Os02g0106900 | LOC_Os02g01730 | Serine/threonine-protein kinase At1g18390 precursor, putative, expressed | 5.78 | 1.02E-14 | Down |
| Os05g0436100 | LOC_Os05g36050 | Serine/threonine-protein kinase, putative, expressed | 5.71 | 1.42E-02 | Down |
| Os01g0607900 | LOC_Os01g42294 | Inactive receptor kinase At2g26730 precursor, putative, expressed | 5.69 | 1.29E-12 | Down |
| Os05g0369700 | LOC_Os05g30680 | Leucine zipper protein-like, putative, expressed | 5.66 | 1.27E-02 | Down |
| Os11g0620500 | LOC_Os11g40550 | Receptor kinase, putative, expressed | 5.62 | 9.11E-03 | Down |
| Os11g0691800 | LOC_Os11g46960 | Receptor-like protein kinase precursor, putative, expressed | 5.61 | 9.52E-06 | Down |
| Os01g0601200 | LOC_Os01g41730 | Serine/threonine-protein kinase, putative, expressed | 5.54 | 2.05E-02 | Down |
| Os07g0498400 | LOC_Os07g31500 | Leucine-rich repeat receptor protein kinase EXS precursor, putative, expressed | 5.23 | 4.91E-05 | Down |
| Os10g0467900 | LOC_Os10g32990 | Receptor-like protein kinase 2 precursor, putative, expressed | 5.17 | 1.77E-04 | Down |
| Os08g0506100 | LOC_Os08g39570 | Leucine Rich Repeat family protein, expressed | 4.93 | 1.90E-02 | Down |
| Os05g0369500 | LOC_Os05g30660 | Leucine zipper protein-like, putative, expressed | 4.91 | 1.11E-02 | Down |
| Os04g0647900 | LOC_Os04g55420 | Leucine-rich repeat family protein, putative, expressed | 4.89 | 2.69E-18 | Down |
| Os04g0410600 | LOC_Os04g33530 | Ser/Thr protein phosphatase family protein, putative, expressed | 4.70 | 1.43E-05 | Down |
| Os08g0376300 | LOC_Os08g28870 | Receptor-like protein kinase 5 precursor, putative, expressed | 4.67 | 2.48E-03 | Down |
| Os12g0595800 | LOC_Os12g40419 | Protein kinase domain containing protein, expressed | 4.04 | 3.10E-10 | Down |
| Os04g0393900 | LOC_Os04g32310 | Serine/threonine-protein kinase NAK, putative, expressed | 3.81 | 5.34E-09 | Down |
| Os01g0664200 | LOC_Os01g47470 | Serine/threonine-protein kinase, putative, expressed | 3.81 | 3.76E-07 | Down |
| Os12g0454800 | LOC_Os12g26940 | Serine/threonine-protein kinase | 3.71 | 2.83E-03 | Down |
| Os10g0571300 | LOC_Os10g42110 | Protein kinase family protein, putative, expressed | 3.70 | 8.87E-05 | Down |
| Os01g0741200 | LOC_Os01g53840 | Protein kinase family protein, putative, expressed | 3.70 | 1.69E-02 | Down |
| Os06g0288100 | LOC_Os06g18000 | Protein kinase domain containing protein, expressed | 3.61 | 4.63E-24 | Down |
| Os03g0301800 | LOC_Os03g18980 | Kinesin motor domain containing protein, expressed | 3.61 | 3.16E-03 | Down |
| Os11g0445300 | LOC_Os11g25860 | Protein Kinase, putative, expressed | 3.61 | 9.38E-16 | Down |
| Os10g0431900 | LOC_Os10g29620 | Tyrosine protein kinase domain containing protein, putative, expressed | 3.61 | 6.02E-08 | Down |
| Os01g0133900 | LOC_Os01g04230 | Inactive receptor kinase At2g26730 precursor, putative, expressed | 3.60 | 1.62E-04 | Down |
| Os04g0202300 | LOC_Os04g12560 | Receptor-like protein kinase, putative, expressed | 3.52 | 5.43E-07 | Down |
| Os06g0557400 | LOC_Os06g36310 | Receptor-like protein kinase 5 precursor, putative, expressed | 3.47 | 3.96E-06 | Down |
| Os03g0101200 | LOC_Os03g01160 | Protein kinase family protein, putative, expressed | 3.46 | 3.12E-05 | Down |
| Os07g0535800 | LOC_Os07g35140 | Receptor-like serine-threonine protein kinase, putative, expressed | 3.43 | 1.36E-05 | Down |
| Os05g0585800 | LOC_Os05g50830 | Protein kinase family protein, putative, expressed | 3.30 | 8.55E-03 | Down |
| Os04g0689400 | LOC_Os04g59320 | Protein kinase domain containing protein, expressed | 3.28 | 8.06E-03 | Down |
| Os07g0550500 | LOC_Os07g36544 | Serine/threonine-protein kinase receptor precursor, putative, expressed | 3.15 | 2.77E-03 | Down |
| Os01g0668600 | LOC_Os01g47820 | S-locus-like receptor protein kinase, putative, expressed | 3.07 | 9.98E-04 | Down |
| Os04g0540900 | LOC_Os04g45730 | Protein kinase domain containing protein, expressed | 3.06 | 5.67E-03 | Down |
| Os03g0332900 | LOC_Os03g21510 | Inactive receptor kinase At2g26730 precursor, putative, expressed | 3.01 | 2.16E-05 | Down |
| Os01g0821900 | LOC_Os01g60670 | Receptor-like protein kinase precursor, putative, expressed | 2.98 | 1.21E-02 | Down |
| Os02g0807900 | LOC_Os02g56380 | Receptor-like protein kinase | 2.95 | 1.50E-02 | Down |
| Os02g0542500 | LOC_Os02g33780 | Serine-aspartate repeat-containing protein I precursor, putative, expressed | 2.84 | 1.08E-02 | Down |
| Os10g0376200 | LOC_Os10g22980 | Leucine rich repeat domain containing protein, putative, expressed | 2.76 | 1.10E-14 | Down |
| Os11g0121400 | LOC_Os11g02830 | Protein kinase domain containing protein, expressed | 2.71 | 2.98E-09 | Down |
| Os01g0769700 | LOC_Os01g56330 | Receptor-like protein kinase | 2.69 | 2.69E-03 | Down |
| Os06g0557700 | LOC_Os06g36320 | Receptor-like protein kinase 5 precursor, putative, expressed | 2.69 | 1.05E-03 | Down |
| Os03g0637800 | LOC_Os03g43670 | Receptor-like protein kinase | 2.54 | 1.79E-03 | Down |
| Os01g0864700 | LOC_Os01g64490 | Protein kinase domain containing protein, expressed | 2.48 | 6.70E-06 | Down |
| Os04g0119800 | LOC_Os04g02920 | Leucine-rich repeat family protein, putative, expressed | 2.46 | 3.08E-05 | Down |
| Os10g0187500 | LOC_Os10g10870 | Protein kinase family protein, putative, expressed | 2.42 | 1.74E-09 | Down |
| Os05g0318100 | LOC_Os05g25390 | Tyrosine protein kinase domain containing protein, putative, expressed | 2.30 | 1.99E-02 | Down |
| Os10g0405100 | LOC_Os10g26520 | Protein kinase domain containing protein, expressed | 2.29 | 2.45E-05 | Down |
| Os04g0685900 | LOC_Os04g58910 | Receptor protein kinase TMK1 precursor, putative, expressed | 2.26 | 2.52E-06 | Down |
| Os05g0501400 | LOC_Os05g42210 | Serine/threonine-protein kinase receptor precursor, putative, expressed | 2.26 | 9.59E-03 | Down |
| Os04g0475200 | LOC_Os04g39930 | Receptor-like protein kinase, putative, expressed | 2.25 | 7.99E-04 | Down |
| Os04g0548400 | LOC_Os04g46320 | Inactive receptor kinase At2g26730 precursor, putative, expressed | 2.23 | 2.62E-04 | Down |
| Os06g0163000 | LOC_Os06g06760 | Protein kinase, putative, expressed | 2.23 | 5.87E-05 | Down |
| Os06g0291500 | LOC_Os06g18820 | Serine threonine kinase, putative, expressed | 2.19 | 1.70E-02 | Down |
| Os05g0100700 | LOC_Os05g01040 | Serine/threonine-protein kinase, putative, expressed | 2.19 | 9.17E-09 | Down |
| Os01g0709500 | LOC_Os01g51290 | Protein kinase family protein, putative, expressed | 2.19 | 6.95E-08 | Down |
| Os04g0619400 | LOC_Os04g52840 | Tyrosine protein kinase domain containing protein, putative, expressed | 2.19 | 5.91E-04 | Down |
| Os07g0681100 | LOC_Os07g48310 | Inactive receptor kinase At2g26730 precursor, putative, expressed | 2.18 | 7.36E-04 | Down |
| Os07g0194100 | LOC_Os07g09610 | Protein kinase | 2.17 | 1.54E-02 | Down |
| Os02g0799700 | LOC_Os02g55610 | Serine-rich protein, putative, expressed | 2.16 | 5.40E-03 | Down |
| Os11g0232100 | LOC_Os11g12530 | Receptor-like protein kinase 5 precursor, putative, expressed | 2.15 | 1.88E-06 | Down |
| Os03g0717000 | LOC_Os03g50810 | Receptor protein kinase TMK1 precursor, putative, expressed | 2.12 | 1.60E-02 | Down |
| Os10g0101000 | LOC_Os10g01100 | Receptor-like protein kinase, putative, expressed | 2.11 | 5.10E-04 | Down |
| Os01g0239700 | LOC_Os01g13800 | Receptor-like protein kinase 5 precursor, putative, expressed | 2.05 | 2.76E-03 | Down |
| Os11g0678000 | LOC_Os11g45280 | Protein kinase family protein, putative, expressed | 2.04 | 6.45E-04 | Down |
| Os04g0605300 | LOC_Os04g51580 | Leucine rich repeat containing protein, expressed | 2.04 | 1.45E-07 | Down |
| Os06g0587000 | LOC_Os06g38730 | Receptor-like protein kinase precursor, putative, expressed | 2.12 | 3.62E-03 | Up |
| Os10g0394100 | LOC_Os10g25430 | Ser/Thr protein phosphatase family protein, putative, expressed | 2.12 | 2.09E-03 | Up |
| Os08g0521200 | LOC_Os08g40990 | Receptor-like protein kinase 1, putative, expressed | 2.15 | 6.21E-03 | Up |
| Os09g0544300 | LOC_Os09g37230 | Protein kinase family protein, putative, expressed | 2.16 | 6.44E-04 | Up |
| Os02g0191700 | LOC_Os02g09840 | Serine/threonine-protein phosphatase 2A regulatory subunit B subunitbeta, putative, expressed | 2.21 | 1.31E-03 | Up |
| Os02g0473200 | LOC_Os02g27360 | Aspartic proteinase-like protein 2 precursor, putative, expressed | 2.38 | 6.70E-04 | Up |
| Os01g0117700 | LOC_Os01g02840 | Esistance-related receptor-like kinase, putative, expressed | 2.39 | 2.02E-02 | Up |
| Os08g0247600 | LOC_Os08g14940 | Receptor kinase, putative, expressed | 2.62 | 1.32E-08 | Up |
| Os02g0215900 | LOC_Os02g12440 | Leucine-rich repeat receptor protein kinase | 2.71 | 6.45E-03 | Up |
| Os01g0115600 | LOC_Os01g02560 | Ser/Thr receptor-like kinase, putative, expressed | 3.25 | 3.63E-04 | Up |
| Os08g0506400 | LOC_Os08g39590 | Inactive receptor kinase At2g26730 precursor, putative, expressed | 3.43 | 1.50E-04 | Up |
| Os02g0216000 | LOC_Os02g12450 | Receptor-like protein kinase 2 precursor, putative, expressed | 3.80 | 8.47E-04 | Up |
| Os01g0138300 | LOC_Os01g04570 | Ser/Thr protein kinase, putative, expressed | 3.85 | 7.38E-05 | Up |
| Os11g0197000 | LOC_Os11g09110 | Receptor-like protein kinase 5 precursor, putative, expressed | 4.32 | 1.07E-09 | Up |
| Os04g0632600 | LOC_Os04g54010 | Serine/threonine-protein kinase receptor precursor, putative, expressed | 5.10 | 6.52E-06 | Up |
| Os09g0345300 | LOC_Os09g17630 | Receptor-like protein kinase 2, putative, expressed | 5.12 | 2.96E-09 | Up |
| Os04g0420033 | LOC_Os04g34290 | Protein kinase, putative, expressed | 5.19 | 4.93E-16 | Up |
| Os01g0137282 | LOC_Os01g04490 | Ser/Thr protein kinase, putative, expressed | 5.58 | 7.06E-07 | Up |
