# Supplementary material for: Rice Carbohydrate-Binding Malectin-Like Protein, OsCBM1, Contributes to Drought-Stress Tolerance by Participating in NADPH Oxidase-Mediated ROS Production
Source: Rice (N Y). 2021 Dec 7;14:100. doi: 10.1186/s12284-021-00541-5 (PMC8651890; doi:10.1186/s12284-021-00541-5)
Supplement: Supplementary file 8 — Additional file 8: Table S1. The primer sequences used in the study. [file 12284_2021_541_MOESM8_ESM.docx]

**Additional file 8: Table S1**

| **Gene Name** | **Gene_Symbol** | **Primer Sequences** |
| --- | --- | --- |
| **Real-time PCR** | | |
| *OsActin1* | LOC-Os03g50885 | F: GTGGTCGCCCCTCCTGAAAG |
|  |  | R: GGCTTAGCATTCTTGGGTCCG |
| *OsCBM1* | LOC_Os09g19400 | F: CATTGGGACGAACTACTGGG |
|  |  | R: AGAAACACCGCGAACTGGC |
| OsMAPKKK1 | LOC_Os03g06410 | F: GCAGTCGGCTTCCAGGACAA |
|  |  | R: TGCAAACGAGGGCCGCAAAT |
| OsMAPKK2 | LOC_Os10g29540 | F: TGTGCAGCACCAGGTCGTAA |
|  |  | R: CAGGCGTTCTCCAGTGGCA |
| OsMAPKKK4 | LOC_Os02g12810 | F: GCAATTGCCGCGCAGGATAG |
|  |  | R: CGGACCGCCCATAGCATCAA |
| OsMAPKKK11 | LOC_Os07g02780 | F: ACGACACCAACTGCTCAAGGTC |
|  |  | R: TCCGCGGAGATGAAGTTGCC |
| OsMAPKKK18 | LOC_Os03g55560 | F: GCGATCTCCTCGCTCCAGTC |
|  |  | R: CCCATTGCCCTCAGGACGAG |
| OsMAPKKK24 | LOC_Os04g56530 | F: CGTGGCACGTGAGTACCTTGA |
|  |  | R: GCCGTGCAAGAAGGCTTTGG |
| OsMKK1 | LOC_Os06g05520 | F: GGCGCCAGAAAGAATCAGTGG |
|  |  | R: GGGTGGTCGACAACAGCTTCA |
| OsMKK3 | LOC_Os06g27890 | F: ATGAAGGCCCAGCCAACCTC |
|  |  | R: AGGCCTTGCATCAGCGTCTT |
| OsMKK4 | LOC_Os02g54600 | F: GCCCTCATGTGCGCGATTTG |
|  |  | R: GGCGACGAACCGATGTTGGA |
| OsMKK5 | LOC_Os06g09180 | F: CATCTGGAGCTTCGGCCTCA |
|  |  | R: GGGAGGGTCGGAATAGCAGA |
| OsMKK6 | LOC_Os01g32660 | F: TGGGTCAGCGGGATACGTTTG |
|  |  | R: GGGAACCGACCAATGGCACA |
| OsMAPK3 | LOC_Os03g17700 | F: TACGGGATCGTCTGCTCCGT |
|  |  | R: TCGAGGTGCCTGAGGAGCTT |
| OsMAPK4 | LOC_Os10g38950 | F: CCGCAACATGTCTGCTGGTG |
|  |  | R: GCTGGGCAGGTGGGTTCTTC |
| OsMAPK6 | LOC_Os06g06090 | F: CCGAGACTTGAAGCCCAGCA |
|  |  | R: CAACAGAAGTTCCGGTGCCCTA |
| OsMAPK7 | LOC_Os06g48590 | F: TGCTCTGCTGCGACAATTAC |
|  |  | R: GCCAAGAAGCTCAGCAAAGA |
| OsMAPK14 | LOC_Os02g05480 | F: CTTTGCGGGAGCTGAAACTT |
|  |  | R: TGGAAAGACCCTGAGGTGAC |
| OsRacGEF1 | LOC_Os09g37270 | F: GGATCGTGGTTGATGACAGC |
|  |  | R: CAATCCCTGTCCTGTTGCAG |
| OsRacGEF2 | LOC_Os05g48640 | F: AGTCCACCCTTGGAGATTCA |
|  |  | R: CCAAATGACCACAGAGGCTT |
| OsRacGEF3 | LOC_Os02g17240 | F: ACATGGACAACTTCGTGGAC |
|  |  | R: TTCCTCGTCACCTCCGATAG |
| OsRacGEF4 | LOC_Os02g47420 | F: CCTCAAAGCTGCGATGTCAA |
|  |  | R: CAGCTCTGCCAGACTTAGGA |
| OsRacGEF5 | LOC_Os01g62990 | F: GTGACAAAGCGAGGAAGCAA |
|  |  | R: ATTGCTGTTGATGGCCAAGG |
| OsRacGEF6 | LOC_Os01g48410 | F: ATCAAGGAGAAGGCCCAGTC |
|  |  | R: TGTCCTCGTGTTCTTGCTCT |
| OsRacGEF7 | LOC_Os10g40270 | F: AACCTCTACGCTAATGCAGC |
|  |  | R: CGATGTAGTCGCATACGGAG |
| OsRacGEF8 | LOC_Os01g55520 | F: CTCGTCTGATCTCTGCTCGT |
|  |  | R: TCCGAGCACGAGTCGCGCTT |
| OsRacGEF9 | LOC_Os04g47170 | F: GAGCTGTACAAGCAAACGCT |
|  |  | R: GGTACTCCGGCGAGAACTT |
| OsRacGEF10 | LOC_Os05g38000 | F: ACACGGAGAAGAGAGACGAC |
|  |  | R: CTCCAGAACCCTCGAGTAGC |
| OsRacGEF11 | LOC_Os07g29780 | F: AGACTACCCTGGACACAAGC |
|  |  | R: TTTCCAGCACCCTCGAGTAG |
| OsRac1 | LOC_Os01g12900 | F: TAGTACAGCAACCAGCAAGAACAAA |
|  |  | R: ACTTGATGAACCTCGTCGCC |
| OsRac2 | LOC_Os05g43820 | F: GCTGCTTGTCTCTGTTCGCC |
|  |  | R: CGCTCATCTCGCCTACTAGGAC |
| OsRac3 | LOC_Os02g50860 | F: CCTCCTCCCATCTTGTCTCCT |
|  |  | R: TCACTCCCGTTTCACCGAG |
| OsRac4 | LOC_Os06g12790 | F: CGCTTCACCAAGAAGCCATC |
|  |  | R: GCCGGCCGAATCGAG |
| OsRac5 | LOC_Os02g58730 | F: ACCGGATGCGCTGGC |
|  |  | R: ACGGTGACGCACTTGATGAAC |
| OsRac6 | LOC_Os02g02840 | F: TGCGGCAATAAAGGTGGTG |
|  |  | R: CCCCTTTGCGCCTTTTTC |
| OsRac7 | LOC_Os02g20850 | F: CAACCAAGAACAAGTTTTCAGGG |
|  |  | R: CGGTGACGCACTTGATGAAC |
| OsRbohA | LOC_Os01g53294 | F: ATCCGCAAAATAAGCACCTCT |
|  |  | R: CAGTAGCCCATCACATCAAAGAC |
| OsRbohB | LOC_Os01g25820 | F: GGCTTCAATGCCTTCTGGT |
|  |  | R: ATGGCTCCTAAACAACCGA |
| OsRbohC | LOC_Os05g45210 | F: CCAGTGGGTGGGAAAAGTG |
|  |  | R: GTCCGATTGGCGGGTAAA |
| OsRbohD | LOC_Os05g38980 | F: CACAAGGTTATCGCACTGACG |
|  |  | R: AGCGATGAGTATGTTGGTTGA |
| OsRbohE | LOC_Os01g61880 | F: TCAAGGCAGCGATTTACCC |
|  |  | R: CTCGCAAGCCTTCCCAAA |
| OsRbohF | LOC_Os08g35210 | F: CCTTTCTCCATCACTTCAGCA |
|  |  | R: GGGCCATCTACAAGCAACC |
| OsRbohG | LOC_Os09g26660 | F: GTCAAATGCTTATGCTGTCA |
|  |  | R: TGTCCAGTCTCCGTTTGTT |
| OsRbohH | LOC_Os12g35610 | F: TACTTCGGGCAGACACGGAT |
|  |  | R: GCGGGTTGCTGTCACTAAG |
| OsRbohI | LOC_Os11g33120 | F: ACCTTACCTGCGATTTTCCA |
|  |  | R: ACGAAGCAGTGGTGGGAGT |
| **Vector construction** | | |
| p1301-CBM1-OE | F: GGATCCATGACTGCATATGCAGGTTTCTT | |
|  | R: GGATCCAATAAGACGAACATCTATTCTAC | |
| p1301-CBM1-RNAi-S | F: CTCGAGGGTGCCTACACGGACATTGAT | |
|  | R: GAATTCGCCTCAGCTCAACCGACGAC | |
| p1301-CBM1-RNAi-A | F: TCTAGAGGTGCCTACACGGACATTGAT | |
|  | R: AAGCTTGCCTCAGCTCAACCGACGAC | |
| p1300-CBM | F: CGGGATCCCGATGACTGCATATGCAGGTTTC | |
|  | R: GACTAGTAATAAGACGAACATCTATTCTAC | |
| p1301-CBM1 | F: CGGGATCCCGATGACTGCATATGCAGGTTTC | |
|  | R: GACTAGTAATAAGACGAACATCTATTCTAC | |
| pPR-SUC-CBM1 | F: CAATCTATTTTATGTAATGGCCATTACGGCCATGACTGCATATGCAGGTTTC | |
|  | R: TATCGAATTCCTGCAGATGGCCGAGGCGGCCTTAATAAGACGAACATCTATTCTAC | |
| pET32a-CBM1 | F: CGGAATTCCATATGATGACTGCATATGCAGGTTTCTTGAG | |
|  | R: CCGGTCGACAATAAGACGAACATCTATTCTAC | |
| JW772-CBM1 | F: GGGGTACCATGACTGCATATGCAGGTTTCTTGAG | |
|  | R: ACGCGTCGACACTAATAAGACGAACATCTATTCTAC | |
| pBT3-STE-GEF | F: GGTAATGGCCATTACGGCCATGGCGAGCGCGTCGGAGGAC | |
|  | R: GCAGATGGCCGAGGCGGCCTCTCTTTCAGGGGCATCTCCT | |
| p1300-GEF | F: CGGGATCCCGATGGCGAGCGCGTCGGAGGAC | |
|  | R: GACTAGTGTCTCTTTCAGGGGCATCTCC | |
| p1301-GEF | F: TCTAGAATGGCGAGCGCGTCGGAGGACGAC | |
|  | R: GGTACCGTCTCTTTCAGGGGCATCTCCTG | |
| pMAL-GEF | F: CGGAATTCCATATGATGGCGAGCGCGTCGGAGGACG | |
|  | R: CCGGTCGACGTCTCTTTCAGGGGCATCTCCTG | |
| JW771-GEF | F: GGGGTACCATGGCGAGCGCGTCGGAGGAC | |
|  | R: ACGCGTCGACGTCTCTTTCAGGGGCATCTC | |
| pBT3-N-RbohA | F: CTGCAGGGCCATTACGGCCATGCGTGGTGGCGCAAGTTCAGG | |
|  | R: CCATGGGGCCGAGGCGGCCTTGAAATGCTCCTTATGGAATTCGA | |
| p1300-RbohA | F: CGGGATCCCGATGCGTGGTGGCGCAAGTTCAG | |
|  | R: GACTAGTCGAAATGCTCCTTATGGAATTCGA | |
| pMAL- RbohA | F: CGGAATTCCATATGATGCGTGGTGGCGCAAGTTCAG | |
|  | R: CCGGTCGACGTTTACGTAAACCCGCCAGATTCT | |
| JW771-RbohA | F: GGGGTACCATGCGTGGTGGCGCAAGTTCAG | |
|  | R: ACGCGTCGACGAAATGCTCCTTATGGAATTCGA | |
