# Supplementary material for: Rice Carbohydrate-Binding Malectin-Like Protein, OsCBM1, Contributes to Drought-Stress Tolerance by Participating in NADPH Oxidase-Mediated ROS Production
Source: Rice (N Y). 2021 Dec 7;14:100. doi: 10.1186/s12284-021-00541-5 (PMC8651890; doi:10.1186/s12284-021-00541-5)
Supplement: Supplementary file 9 — Additional file 9. Supporting experimental procedures Methods S1. [file 12284_2021_541_MOESM9_ESM.docx]

**Additional file 9: Supporting experimental procedures Methods S1**

**Plant materials and growth conditions**

*Oryza sativa subsp. Japonica* Nipponbare was used as the wild-type (WT) plant in all the experiments. The experiments were performed in the State Key Laboratory of Crops Stress Biology for Arid Areas, Northwest A&F University, Yangling, China. First of all, rice seeds were sterilized with 0.5% (w/v) sodium hypochlorite (NaClO) for 4 h. After washed thrice with distilled water, the seeds were soaked in water for 48 h in darkness and then germinated on humid cheesecloth at 28 °C for 72 h. Subsequently, the healthy and uniform seedlings were selected and grown in hydroponic solution prepared in Milli-Q water (Hoagland and Arnon 1950), containing 16 mM KNO_3_, 6 mM Ca(NO_3_)_2_·4H_2_O, 4 mM NH_4_H_2_PO_4_, 2 mM MgSO_4_·7H_2_O, 50 μM KCl, 25 μM H_3_BO_3_, 25 μM Fe-EDTA, 2 μM MnSO_4_·4H_2_O, 2 μM ZnSO_4_, 0.5 μM Na_2_MoO_4_·2H_2_O, and 0.5 μM CuSO_4_·5H_2_O. The nutrient solutions were continuously aerated and the environment was firmly controlled in growth chamber condition at 16 h/8 h day/night, temperature cycle of 30 °C /25 °C, 800 μmol·m^-2^·s^-1^ light intensity and 60-65% relative humidity level. The solution was changed after 24 h duration and the pH was adjusted to 5.8 by using NaOH or HCl.

For drought sensitivity assessment of plants under soil, seeds were transferred into plastic pots with Hoagland’s nutrient solution after WT, RNAi and OE lines germination. When the fourth leaf had fully expanded (4-week old), the rice seedlings were transferred to plastic pots (soil) in a greenhouse with each pot filled by same weight soil from paddy fields. The cultivated conditions were the same in our previous study (Wang et al. 2016). After two weeks, the plants were subjected to drought stress by withholding water up to 15 days and keeping-watered plants served as controls. After soil drought treatment, watering was resumed and the plants were allowed to grow for seven additional days.

*Nicotianna benthamiana* was also used in this experiment. Seeds of the *N. benthamiana* were germinated on sterile 1/2 MS solid medium at 28 °C for 7 days. Healthy and uniform seedlings were selected and grown in plastic pots, which were each filled with substrate (Growing Media Europe AISBL, Belgium) placed in growth chamber condition at 12 h/12 h day/night, 28°C, 600 μmol·m^-2^·s^-1^ light intensity and 60-75% relative humidity level. Four-week old seedlings were used for the following experiments including the analysis of protein subcellular localization, visualization of ROS production, assays of the firefly luciferase complementation imaging (LCI) and Immunoprecipitation (Co-IP).

**Expression profile analysis**

The expression profiles of *OsCBM1* in different plant organs or tissues and under different environmental conditions were analyzed as described previously (Wang et al., 2016). For the tissue-specific expression analysis, the wild-type of rice plants were allowed to grow in paddy field and the different plant organs or tissues at various developmental stages, namely seedling, tillering, booting and heading stages, were collected. After harvested, the samples were immediately frozen in liquid nitrogen and stored at -80 °C until for further analysis. To identify the inducible expression profiles of *OsCBM1*, the young seedlings (two-week-old) of WT plants grown in growth chamber as described above were exposed to various abiotic stresses and phytohormones. The treatments were carried out as described previously with a little modification (Muhammad et al. 2018). For cold stress, the seedlings were transferred into a cold cabinet (SANYO) under a 14h/10 h light/dark cycle with light conditions of 300 μmol·m^-2^·s^-1^ intensity. For heat stress, the seedlings were subjected at 40°C temperature with 60% humidity, 16 h photoperiod in a growth chamber under fluorescent light for 24 h. For oxidative stress, the seedlings were sprayed with 15 μM methyl viologen (MV). For dehydration treatment, the seedlings were grown in 20% polyethylene glycol (PEG-6000) solution which was purified by passing it through an ion exchange column to remove any impurities and was filtered using Miracloth (22-25 μm, Thomas Scientific, Swedesboro, NJ, USA). For salt stress, the seedlings were grown in 200 mM NaCl solution. For phytohormonal treatments, the final hormonal concentration of abscisic acid (ABA) (100 μM), methyl jasmonate (MeJA) (100 μM), gibberellin (GA) (50 μM) and salicylic acid (SA) (500 μM) were prepared from stock solutions, after addition of wetting agent Tween-20 at 0.05% (v/v) the individual hormone were sprayed on two weeks old rice leaves.

Total RNA was extracted from different kinds of plant tissues by using RNeasy Plant Mini Kit (Qiagen, Dusseldorf, Germany) according to the manufacturer’s protocol. The first strand of cDNA was synthesized from 2 μg of total RNA in 25 µL reaction system using the M-MLV First Strand Kit (Invitrogen, Hangzhou, China). To measure the expression level of *OsCBM1* and other target genes, real-time qRT-PCR was performed with SYBR Es Mix (Roche, Basel, Switzerland) on the Bio-Rad CFX96 Real-Time PCR System (Bio-Rad, Hercules, California, America). The relative expression level of the genes was obtained by normalization to reference gene rice *OsActin1* (Gene ID: KC140126). Three biological replicates with three technical repeats each were performed for each treatment. The mean and SD were calculated from the biological replicates (n = 9). All the RT-PCR and qRT-PCR primers used in this study are listed in Supplemental Table S1.

**Subcellular location analysis**

Subcellular localization of OsCBM1 was determined using a *N. benthamiana* transient transformation system (Chen et al., 2009). The open reading frame (ORF) of *OsCBM1* was fused upstream of the green fluorescent protein (GFP) gene and inserted into the binary vector pCAMBIA1301 to generate an *OsCBM1*-*GFP* plasmid under the control of the constitutive *cauliflower mosaic virus* 35S (CaMV 35S) promoter. The transformation vector was introduced into *Agrobacterium* tumefaciens strain GV3101 and then used to transform the protoplasts of *N. benthamiana*. AtCBL1n-mCherry was served as a marker for PM protein localization (Batistic et al., 2010) and AtCHS-mCherry was used as a marker for ER protein localization (Dana et al., 2006). The co-localization of OsCBM1 with OsRacGEF1 in subcellular compartment was detected using the agrobacterium-mediated transformation with both *N. benthamiana* epidermal cells and protoplasts. Where, the open reading frame (ORF) of *OsRacGEF1* was fused upstream of the red fluorescent protein (mCherry) gene and inserted into the binary vector pCAMBIA1301 to generate an *OsRacGEF1*-*mCherry* plasmid under the control of CaMV 35S promoter. The vectors containing both the *OsCBM1-GFP* and *OsRacGEF1*-*mCherry* fusion genes were then co-transformed into *N. benthamiana*. For the analysis of OsRacGEF1 localization in the *OsCBM1*-RNAi transgenic plants, the *OsRacGEF1*-*mCherry* containing vector was transformed into rice protoplasts from 7-10 day old rice wild type (WT) and *OsCBM1*-RNAi54 seedlings respectively, for about 12-16 h. The fluorescence was observed under a confocal microscope (A1R, Nikon, Tokyo, Japan). The primers used for the generation of the plasmids are listed in Supplemental Table S1.

**Generation of transgenic plants and their phenotypes**

To construct the *OsCBM1*-overexpressing (OE) transgenic lines, the ORF sequence of *OsCBM1* was amplified and then inserted into the binary vector pCAMBIA1301 to generate an overexpressing plasmid under the control of the CaMV 35S promoter. To construct the *OsCBM1*-RNA interference (RNAi) transgenic lines, the partial ORF sequence of *OsCBM1* was amplified and then connected both the forward and reverse sequences to the two ends of intron on the vector pTCK303. The constructs were then introduced into *Agrobacterium tumefaciens* strain EHA105 and then infect callus of WT of rice (cv. Nipponbare). Transgenic rice plants were generated as described previously (Toki et al., 2006). The expression levels of *OsCBM1* in the transgenic lines were detected by real-time qRT-PCR with WT as the control. The primers used for the generation of the plasmids for transgenic plant are listed in Supplemental Table S1. Agronomic traits of the OE, RNAi and WT were measured on 30 plants grown in field at ripening stage for each type plant.

**Analysis of stomata aperture**

At least 1000 stomata taken from six different leaves were photographed with a [scanning electron microscope](http://www.baidu.com/link?url=HDEIAAr3sx7I3FO4KEB78y4Y3kb6iqjeM8Oj3nRF2swtPGrvmh0HfgD-qse1OD5Vs7S8hpgr2rUSOfki8q4BacB9znckuVIyKw0OYTuU64DQPBNJBT4tRi_2-tOMHJ1SXjn8njGwDWL_Lh7rseDQfa) (SU8010, Hitachi, Japan) to study the stomata aperture in the OE, RNAi and WT plants. Strips of abaxial epidermis of the rice leaves were used for the analysis. The leaf materials were immediately fixed by 3% glutaraldehyde in 0.1 M phosphate buffer (pH 7.4) at 4^°^C for 5 h, followed by washing twice with 0.1 M phosphate buffer for 10 min. Subsequently, these samples were fixed by 1% osmium tetroxide in 0.1 M phosphate buffer (pH 7.4) at 4^°^C for 2 h and then washed twice with 0.1 M phosphate buffer for 10 min. After dehydrated serially in 40, 50, 60, 70, 80, 90, and 100% ethanol solution for 20 min each, the samples were dried by critical-point dryer and coated with gold and monitored by the scanning electron microscopy. The status of 100 stomata was analyzed in Image J software by measuring the width and length of stomata in the leaves for each type of plants (Zhang et al. 2011).

**Drought treatment and determination of related parameters**

The well germinated seeds of the OE, RNAi and WT plants as described above were sown in plastic pots filled with Sunshine MVP potting soil (Yangling, China). After two weeks, the seedlings were transferred into other plastic pots with filled by an equal amount of paddy soils for 4 weeks. And then, the plants were started to drought stress treatment by withholding water with the well-watered plants served as controls. After 10 days, the last fully expanded leaves both under normal growth and drought stress conditions were collected for the determination of ROS production, photosynthetic characteristics, and gene expression profiles. Rates of photosynthesis and transpiration, stomatal conductance and water use efficiency were measured with a Li-6400 portable photosynthesis system (Li-COR Biosciences, Lincoln, NE, USA), as described by Wu et al. (2011). The transcription profiles of differentially expressed genes between the OsCBM1-transgenic plants and WT under drought were analyzed with qRT-PCR as described above. For the assay of drought tolerance between the OE, RNAi and WT plants, the 4-week old seedlings grown in Sunshine MVP potting soils were directly treated by withholding water. After 5 days, when most of the plants were clearly withered, watering was resumed for initiating the recovery period. Survival rates of the plants were recorded after 14 days of watering. The drought stress experiment was performed at least three biological replicates.

**Detection of ROS production**

Histochemical analysis of ROS (H_2_O_2_ and O_2_^–^) production in leaves of the OE, RNAi and WT plants were carried out as described previously (Wang et al. 2016). The detached leaves from 6-week old plants grown in normal growth conditions were used for the histochemistry assay. H_2_O_2_ and O_2_^–^ releases in the leaves were visually detected by staining with 3,3’-diaminobenzidine tetrachloride (DAB) and nitro blue tetrazolium (NBT), respectively. The leaves were put into DAB or NBT staining solutions in darkness at room temperature for 8 h. Then, the leaves were fixed with a solution of 3:1:1 ethanol/acetic acid/glycerol. After fixed, the samples were immersed into bleaching solution (75% ethanol). The bleaching process was repeated 2~3 times for the clearer photographs.

Contents of H_2_O_2_ in leaves of different types of the rice plants were determined with a Hydrogen Peroxide Assay Kit (Solarbio Institute of Biotechnology, Beijing, China) by following the manufacturer’s instructions. Samples were ground to homogenate with 1 ml reagent I per 100 mg of tissue. After centrifuged at 8,000 g for 10 min at 4^°^C, the resulted supernatants were used for the next reaction. Mixed 250 μl of the supernatants, 25 μl reagent II and 50 μl reagent III for each test wells, and centrifuged at 4,000 g for 10 min at room temperature. The sediment was used for next reaction. Add 250 μl reagent IV to dissolve the sediment, and stored at room temperature for 5 minutes. The resulted solutions were used for the subsequent measurement of H_2_O_2_ content. 200 μl of the resulted solutions were used for each test well. The absorbance at 415 nm was recorded and the contents of H_2_O_2_ were calculated according to a standard curve.

O_2_^–^ production rate was measured as described by Jiang and Zhang (2003) by monitoring the reduction of sodium 3’-[1-[phenylamino-carbonyl]-3,4-tetrazolium]-bis(4-methoxy-6-nitro) benzenesulfonic acid hydrate (XTT) in the presence of O_2_^–^ with some modifications. Leaf samples (0.4 g) were homogenized with 2 ml of extract solution (50 mM Tris-HCl, pH7.5) and then centrifuged at 3,000 g for 10 min at 4°C. The reaction mixture (1 ml) contained 50 mM Tris-HCl buffer (pH 7.5), 50 mg leaf supernatant proteins and 0.5 mM XTT. The reduction of XTT was determined at 470 nm for 5 min. Corrections were made for the background absorbance in the presence of 50 units SOD. The production rate of O_2_^–^ was calculated using an extinction coefficient of 2.16*10^4^ M^-1^ cm^-1^.

Visualization of ROS *in situ* in *N. benthamiana* leaves was performed as described by Wong et al. (2007). *Agrobacterium tumefaciens strain* GV3101 harboring plasmids pCAMBIA1300-221-cMyc vector carrying the ORF of *OsCBM1*, *OsRacGEF1* or the empty vector were used to infiltrate 4-week old *N. benthamiana* leaves. The agroinfiltrated plants were kept in a growth chamber at 75% humidity for 2 d after agroinfiltration. The agroinfiltrated leaves were picked and dyed in the same way as the rice leaves. DAB-stained leaves were scanned, and pixel intensity of agroinfiltrated regions was quantified by the Image J (National Institutes of Health) software. The mean pixel intensity from three spots outside the infiltrated regions of each leaf was used for background subtraction, and main veins were avoided in all regions selection. The relative DAB stain intensity was calculated based on the pixel intensity of the control cMyc-agroinfiltrated region of each leaf to facilitate comparison of the DAB staining between different treatments. The agroinfiltrated leaves with different vectors were also used to isolate protoplasts according to the described by Zhang et al. (2011). ROS production in the protoplasts was visualized via fluorescence of H_2_DCFDA as described previously (Wang et al., 2016). The intensity of florescent signals was calculated with Image J1.8.0 software and presented with scatter diagrams.

**Pull-down assay**

The protein expression in vitro and purification were carried out as described by Chen et al. (2009). In the interaction analysis of OsRacGEF1 and OsCBM1, the Full-length coding sequence of *OsRacGEF1* was amplified and ligated into the pMAL-c2X vector for the preparation of recombinant MBP-OsRacGEF1 protein and the coding sequence of *OsCBM1* was amplified and ligated into the pET-32a vector for the preparation of recombinant His-OsCBM1 protein (Finkelstein and Lynch, 2000; Nakamura et al., 2001). In the interaction analysis of OsRbohA and OsCBM1, the NADPH-Ox domain at the N-terminal of *OsRbohA* (coding 360 amino acid residues at the N-terminal) was amplified and ligated into the pMAL-c2X vector for the preparation of the recombinant MBP-OsRbohA-ND protein, and the coding sequence of *OsCBM1* was amplified and ligated into the pET-32a vector for the preparation of the recombinant His-OsCBM1 protein. The constructs were transformed into *Escherichia coli* strain BL21. Expression was induced with isopropyl b-D-1-thiogalactopyranoside (IPTG), and the recombinant proteins were purified according to the manufacturer’s protocols (GE). Protein concentration was measured using the Protein Assay Kit II (Bio-Rad). The pull-down assay were performed according to the method described by Zhang et al. (2010). The primers used for the generation of the recombinant proteins are listed in Supplemental Table S1.

**Split-ubiquitin yeast two-hybrid assays**

The full-length coding sequences of OsRacGEF1 and OsCBM1 were amplified and cloned into vectors pBT-STE and pPR-SUC, respectively. The plasmids were co-transformed into *Saccharomyces cerevisiae* NMY51 yeast cells and grown on SD/-Leu-Trp medium. Further, the yeast cells were screened on the SD/-Leu-Trp-His-Ade medium. The performance for protein interaction analysis was carried out according to the Yeast Protocols Handbook (Clontech, Mountain View, CA, USA). The primers used for generating the various vectors are listed in Supplemental Table S1.

**Firefly luciferase complementation imaging (LCI) assay**

The LCI assays for the protein interaction detection were performed in *N. benthamiana* leaves as described previously (Sun et al., 2013). In the interaction analysis of OsRacGEF1 and OsCBM1, the full-length of coding regions of *OsRacGEF1* and *OsCBM1* were fused with the N- and C-terminal parts of the luciferase reporter gene LUC, respectively. In the interaction analysis of OsRbohA and OsCBM1, the full-length of coding regions of *OsRbohA* and *OsCBM1* were fused with the C- and N-terminal parts of the luciferase reporter gene LUC, respectively. Agrobacteria harboring nLUC and cLUC derivative constructs were coinfiltrated into *N. benthamiana*, and the infiltrated leaves were analyzed for LUC activity at 48 h after infiltration using a plant living molecular marker imaging system (Lumazone Pylon 2048B, Princeton, American). Three biological replicates were performed with similar results. The list of primer sets used for the generation of the constructs are listed in Supplemental Table S1.

**Immunoprecipitation assay**

The Co-immunoprecipitation (Co-IP) assay was carried out with described previously (Zhang et al., 2018). The Full-length coding sequence of *OsRacGEF1* was amplified and ligated into the pCAMBIA 1300-221-6×cMyc vector for the preparation of recombinant cMyc-OsRacGEF1 protein. The Full-length coding sequence of *OsCBM1* was fused upstream of the green fluorescent protein (GFP) gene and inserted into the binary vector pCAMBIA1301-eGFP for the preparation of recombinant OsCBM1-eGFP proteins. The transformation vectors were introduced into *Agrobacterium tumefaciens strain* GV3101 and then used to transform the leaves of *N. benthamiana*. The total infected leaves were homogenized in protein lysis buffer (50 mM Tris-HCl at pH 7.5, 150 mM NaCl, 5 mM EDTA at pH 8.0, 0.1% Triton X-100, 0.2% Nonidet P­40, 10 mM PMSF, and 20 mM MG132 with Roche protease inhibitor cocktail). After protein extraction, 20 μL protein G plus agarose were added to reduce nonspecific immunoglobulin binding. Centrifuged and retained supernatants after 1 h of incubation, and cMyc antibody-bound agarose beads were then added to each reaction and incubated 2h at 4°C. Then the cMyc antibody-bound agarose beads were washed at three times using the lysis buffer. Anti-GFP (1:2000; CWBIO, China) and anti-cMyc (1:2000; CWBIO, China) antibodies were used for the detection of GFP-tagged proteins with western blotting. Three independent biological replicates were performed for the analysis. The primer sets used for the generation of the constructs are listed in Supplemental Table S1.

**RNA-seq Analysis**

For transcriptional analysis, the leaves of 6-week old plants of *OaCBM1*-RNAi54 and WT (cv. Nipponbare) plants grown in plastic pots with filled by paddy soil were harvested and stored immediately in liquid nitrogen. Three biological replicates were used, and each replicate was comprised of leaf tissues from more than 30 plants. Total RNA of each sample was extracted using RNeasy Plant Mini Kit (Qiagen 74904, Dusseldorf, Germany). Next generation sequencing library preparations were constructed according to the manufacturer’s protocol (NEBNext® Ultra™ RNA Library Prep Kit for Illumina®). Libraries with different indexes were multiplexed and loaded on an Illumina HiSeq instrument according to manufacturer’s instructions (Illumina, San Diego, CA, USA). Sequencing was carried out using a 2x150bp paired-end (PE) configuration; image analysis and base calling were conducted by the HiSeq Control Software (HCS)+OLB+GAPipeline-1.6 (Illumina) on the HiSeq instrument. The sequences were processed and analyzed by GENEWIZ, Inc., Suzhou, China. Only genes with associated transcript levels that had increased or decreased more than twofold and had associated p-values of <0.05 were used for the further analysis.

**Statistical analyses**

Student’s t-tests were performed using SPSS 11.5 software (SPSS, Inc., Chicago, IL). Differences were considered significant at p ≤ 0.05 based on a one-way analysis of variance (ANOVA).

**References**

**Batistic O, Waadt R, Steinhorst L, Held K, Kudla J** (2010) CBL-mediated targeting of CIPKs facilitates the decoding of calcium signals emanating from distinct cellular stores. Plant Journal **61**: 211–222.

**Chen YF, Li LQ, Xu Q, Kong YH, Wang H, Wu WH (2009)** The WRKY6 transcription factor modulates PHOSPHATE1 expression in response to low Pi stress in Arabidopsis. Plant Cell **21**: 3554-3566.

**Dana CD, Bevan DR, Winkel** BSJ (2006) Molecular modeling of the effects of mutant alleles on chalcone synthase protein structure. Journal of Molecular Modeling **12**: 905-914.

**Finkelstein RR, Lynch TJ** (2000) The Arabidopsis abscisic acid response gene ABI5 encodes a basic leucine zipper transcription factor. Plant Cell **12**: 599–609.

**Jiang M, Zhang J** (2003) Cross-talk between calcium and reactive oxygen species originated from NADPH oxidase in abscisic acid-induced antioxidant defence in leaves of maize seedlings. Plant, Cell & Environment, **26**: 929-939.

**Muhammad I, Jing X-Q, Shalmani A, Muhammad A, Shi Y, Gan P-F, Li W-Q, Liu W-T, Chen K-M** (2018) Comparative in Silico Analysis of Ferric Reduction Oxidase (FRO) Genes Expression Patterns in Response to Abiotic Stresses, Metal and Hormone Applications. Molecules, **23**: 1163.

**Nakamura S, Lynch TJ, Finkelstein RR** (2001) Physical interactions between ABA response loci of Arabidopsis. The Plant Journal **26**: 627-635.

**Toki S, Hara N, Ono K, Onodera H, Tagiri A, Oka S, Tanaka H** (2006) Early infection of scutellum tissue withAgrobacterium allows high-speed transformation of rice. The Plant Journal **47**: 969–976.

**Wang X, Zhang M-M, Wang Y-J, Gao Y-T, Li R, Wang G-F, Li W-Q, Liu W-T, Chen K-M** (2016) The plasma membrane NADPH oxidase OsRbohA plays a crucial role in developmental regulation and drought-stress response in rice. Physiologia Plantarum **156**: 421-443.

**Wong HL, Pinontoan R, Hayashi K, Tabata R, Yaeno T, Hasegawa K, Kojim C, Yoshioka H, Iba K, Kawasaki T, Shimamoto K** (2007) Regulation of rice NADPH oxidase by binding of Rac GTPase to its N-terminal extension. The Plant Cell **19**: 4022-4035.

**Wu L-L, Liu Z-L, Wang J-M, Zhou C-Y, Chen K-M (2011)** Morphological, anatomical, and physiological characteristics involved in development of the large culm trait in rice. Australian Journal of Crop Science 5: 1356-1363.

**Zhang M, Chiang Y-H, Toruño TY, Lee DH, Ma M, Liang X, Lal NK, Lemos M, Lu Y-J, Ma S, Liu J, Day B, Dineshi-Kumar SP, Dehesh K, Dou D, Zhou J-M, Coaker G** (2018) The MAK4 kinase SIK1 ensures robust extracellular ROS burst and antibacterial immunity in plants. Cell Host & Microbe **24**: 1-13.

**Zhang Y, Su J, Duan S, Ao Y, Dai J, Liu J, Wang P, Li Y, Liu B, Feng D, Wang J, Wang H** (2011) A highly efficient rice green tissue protoplast system for transient gene expression and studying light/chloroplast-related processes. Plant Methods **7**: 30.

**Zhang J, Li W, Xiang T, Liu Z, Laluk K, Ding X, Zou Y, Gao M, Zhang X, Chen S** (2010) Receptor-like Cytoplasmic Kinases Integrate Signaling from Multiple Plant Immune Receptors and Are Targeted by a Pseudomonas syringae Effector. Cell Host & Microbe, **7**: 290-301.
